# Selective Pressures to Maintain Attachment Site Specificity of Integrative and Conjugative Elements

**DOI:** 10.1371/journal.pgen.1003623

**Published:** 2013-07-18

**Authors:** Kayla L. Menard, Alan D. Grossman

**Affiliations:** Department of Biology, Massachusetts Institute of Technology, Cambridge, Massachusetts, United States of America; Uppsala University, Sweden

## Abstract

Integrative and conjugative elements (ICEs) are widespread mobile genetic elements that are usually found integrated in bacterial chromosomes. They are important agents of evolution and contribute to the acquisition of new traits, including antibiotic resistances. ICEs can excise from the chromosome and transfer to recipients by conjugation. Many ICEs are site-specific in that they integrate preferentially into a primary attachment site in the bacterial genome. Site-specific ICEs can also integrate into secondary locations, particularly if the primary site is absent. However, little is known about the consequences of integration of ICEs into alternative attachment sites or what drives the apparent maintenance and prevalence of the many ICEs that use a single attachment site. Using ICE*Bs1*, a site-specific ICE from *Bacillus subtilis* that integrates into a tRNA gene, we found that integration into secondary sites was detrimental to both ICE*Bs1* and the host cell. Excision of ICE*Bs1* from secondary sites was impaired either partially or completely, limiting the spread of ICE*Bs1*. Furthermore, induction of ICE*Bs1* gene expression caused a substantial drop in proliferation and cell viability within three hours. This drop was dependent on rolling circle replication of ICE*Bs1* that was unable to excise from the chromosome. Together, these detrimental effects provide selective pressure against the survival and dissemination of ICEs that have integrated into alternative sites and may explain the maintenance of site-specific integration for many ICEs.

## Author Summary

Integrative and conjugative elements (ICEs) are mobile genetic elements that transfer DNA between bacteria, driving bacterial evolution and the acquisition of new traits, including antibiotic resistances. ICEs normally reside integrated in a host genome, but can excise and transfer to recipient cells. Many ICEs are site-specific, predominantly integrating into a single “attachment” site. Others are more promiscuous, capable of integrating into many different sites. Little is known about the consequences of a site-specific ICE integrating into an alternative attachment site, or the selective pressures that maintain the specificity of integration for ICEs with a single attachment site. We found that integration of ICE*Bs1*, a site-specific ICE in *Bacillus subtilis*, into alternative attachment sites was detrimental to both ICE*Bs1* and the host cell. Excision of ICE*Bs1* from alternative attachment sites was reduced or eliminated, thereby limiting mobility of the element. In addition, when ICE*Bs1* gene expression was activated, cell proliferation and viability dropped if ICE*Bs1* was in an alternative attachment site. This drop was due to autonomous replication of ICE*Bs1* that was stuck in the host chromosome. These detrimental effects likely provide strong selective pressure to maintain attachment site specificity of ICE*Bs1* and likely many other site-specific ICEs.

## Introduction

Integrative and conjugative elements (ICEs, also known as conjugative transposons) are mobile genetic elements that encode conjugation machinery that mediates their transfer from cell to cell. Most characterized ICEs were identified because they carry additional genes that confer phenotypes to the host cell. These can be genes involved in pathogenesis, symbiosis, and antibiotic resistances, among others {reviewed in [1]}. ICEs are typically found integrated in the host bacterial chromosome and can excise to form a circular product that is the substrate for conjugation. Their ability to spread to other organisms through conjugation makes ICEs important agents of horizontal gene transfer in bacteria, and they appear to be more prevalent than plasmids [2]. ICEs can also facilitate transfer (mobilization) of other genetic elements [1], [3], [4].

Some ICEs have a specific integration (attachment or insertion) site in the host genome whereas others are more promiscuous and can integrate into many locations. For example, SXT, an ICE in *Vibrio cholera* has one primary site of integration in the 5′ end of *prfC* [5]. In contrast, Tn*916* has a preference for AT-rich DNA in many different hosts and integrates into many different chromosomal sites [6], [7]. Each strategy for integration has its benefits. The more promiscuous elements can acquire a wider range of genes adjacent to the integration sites, and their spread is not limited to organisms with a specific attachment site. On the other hand, site-specific elements are much less likely to disrupt important genes. The attachment site for these elements is typically in a conserved gene, often a tRNA gene [8], [9]. If sequences at the end of the integrating element are identical with the 3′ end of the gene (which is often the case), then gene function is not disrupted. Integration into conserved genes makes it likely that many organisms will have a safe place for these elements to integrate. We wished to learn more about the ability of site-specific ICEs to integrate into secondary integration (or attachment) sites, particularly if the primary site is not present in a genome. We wondered if an ICE could function normally in a secondary site and if there was any effect on the host.

**Figure 1:**
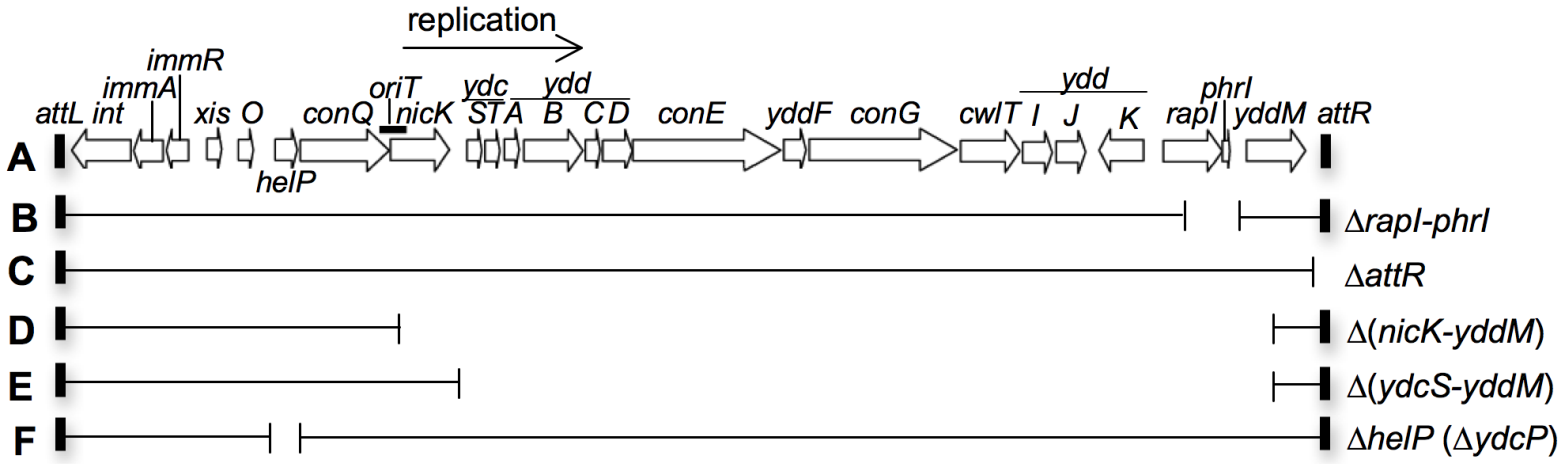
Map of ICE*Bs1* and its derivatives. **A.** The linear genetic map of ICE*Bs1* integrated in the chromosome. Open arrows indicate open reading frames and the direction of transcription. Gene names are indicated above or below the arrows. The origin of transfer (*oriT*) is indicated by a thick black line overlapping the 3′ end of *conQ* and the 5′ end of *nicK*. *oriT* functions as both the ICE*Bs1* origin of transfer and origin of replication [15], [23]. The thin black arrow indicates the direction of ICE*Bs1* rolling-circle replication. The small rectangles at the ends of ICE*Bs1* represent the 60 bp direct repeats that contain the site-specific recombination sites in the left and right attachment sites, *attL* and *attR*, that are required for excision of the element from the chromosome. **B–F.** Various deletions of ICE*Bs1* were used in this study. Thin horizontal lines represent regions of ICE*Bs1* that are present and gaps represent regions that are deleted. Antibiotic resistance cassettes that are inserted are not shown for simplicity. **B.** *rapI* and *phrI* are deleted and a kanamycin resistance cassette inserted. **C.** The right attachment site (*attR*) is deleted and a tetracycline resistance cassette inserted. **D.** The genes from the 5′ end of *nicK* and into *yddM* are deleted and a chloramphenicol resistance cassette inserted. **E.** The genes from the 5′ end of *ydcS* and into *yddM* are deleted and a chloramphenicol resistance cassette inserted. **F.** The entire coding sequence of *helP* (previously known as *ydcP*) and 35 bp in the *helP*-*ydcQ* intergenic region is removed. There is no antibiotic resistance cassette in this construct.

We used ICE*Bs1* of *Bacillus subtilis* to analyze effects of integration into secondary attachment sites. ICE*Bs1* is a site-specific conjugative transposon that is normally found integrated into a tRNA gene (*trnS-leu2*) [10], [11]. ICE*Bs1* is approximately 20 kb (Figure 1), and many of its genes are similar to genes in other ICEs, including those in Tn*916* [11], [12], the first conjugative transposon identified [13], [14]. It is not known what properties or advantages ICE*Bs1* confers on host cells, and naturally occurring ICE*Bs1* is not known to carry genes involved in antibiotic resistances, virulence, or metabolism. However, because of the conservation of many of its functions, the ease of manipulating *B. subtilis*, and the high efficiency of experimental induction of gene expression, ICE*Bs1* is extremely useful for studying basic and conserved properties of ICEs.

Induction of ICE*Bs1* gene expression leads to excision from the chromosome in >90% of the cells, autonomous rolling-circle replication of ICE*Bs1*, and mating in the presence of appropriate recipients [10], [11], [15]. After excision from the chromosome, autonomous replication of ICE*Bs1* is needed for its stability during cell growth and division [15]. In addition, excision is not needed for replication; ICE*Bs1* that is unable to excise from the chromosome undergoes autonomous unidirectional replication following induction of ICE*Bs1* gene expression [15]. At least some other ICEs appear to undergo autonomous replication [1], [16]–[18]. In addition, the genes in ICE*Bs1* that are required for autonomous replication are conserved [19]. Based on these observations and the properties of ICE*Bs1*, we suspect that many ICEs undergo rolling circle replication and use the origin of transfer as an origin of replication and the cognate conjugative relaxase as a replicative relaxase [3], [19].

Our aim was to examine the physiological consequences of integration of ICE*Bs1* into secondary attachment sites. Previous work showed that in the absence of its primary attachment site (*attB* in the gene for tRNA-leu2), ICE*Bs1* integrates into secondary attachment sites [10]. Seven different sites were identified and characterized previously, providing insight into the chromosomal sequences needed for integration [10]. Work presented here extends these findings by identifying additional secondary sites, evaluating the ability of ICE*Bs1* to excise from these sites, and determining the effects of integration at these sites on host cells. Our results indicate that integration of ICE*Bs1* in secondary integration sites is deleterious to ICE*Bs1* and to the host cell. Excision and spread of ICE*Bs1* from the secondary sites was reduced or eliminated and there was a drop in cell viability due to autonomous replication of ICE*Bs1* that was defective in excision. These effects likely provide strong selective pressure for insertions into sites from which ICE*Bs1* can excise and against the propagation of insertions in secondary sites.

## Results

### Identification of secondary sites of integration of ICE*Bs1*

We identified 27 independent insertions of ICE*Bs1* into secondary integration sites in the *B. subtilis* chromosome. Briefly, these insertions were identified by: 1) mating ICE*Bs1* into a recipient strain deleted for the primary attachment site *attB* (located in the tRNA gene *trnS-leu2*), 2) isolating independent transconjugants, and 3) determining the site of insertion in each of 27 independent isolates. The frequency of stable acquisition of ICE*Bs1* by strains missing *attB* was reduced to ∼0.5–5% of that of strains containing *attB* {Materials and Methods, and [Materials and Methods, and 10]}.

**Figure 2:**
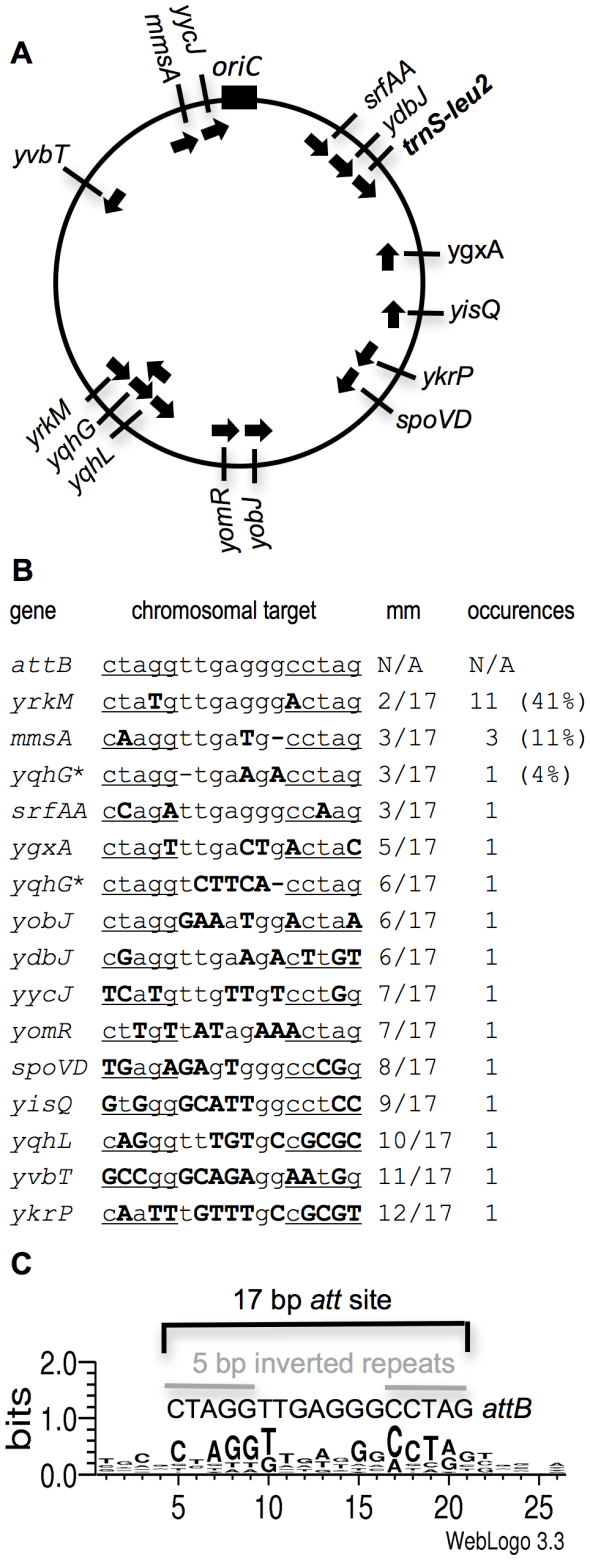
Map and DNA sequence of the primary and 15 secondary integration sites for ICE*Bs1*. **A.** Approximate position of the primary and 15 secondary ICE*Bs1* integration sites on the *B. subtilis* chromosome. The circle represents the *B. subtilis* chromosome with the origin of replication (*oriC*) indicated by the black rectangle at the top. The slash marks represent the approximate location of the ICE*Bs1* insertion site. The name of the gene near which (*ygxA*) or into which (all other locations) ICE*Bs1* inserted is indicated on the outside of the circle. The arrows on the inside of the circle indicate the direction of ICE*Bs1* replication for each insertion. *trnS-leu2* (in bold) contains the primary ICE*Bs1* integration site *attB*. **B.** DNA sequence of the primary and 15 secondary integration sites. The gene name is indicated on the left, followed by the DNA sequence (chromosomal target). The primary attachment site (*attB*) is a 17 bp sequence with 5 bp inverted repeats (underlined) separated by a 7 bp spacer. Mismatches from *attB* are indicated in bold, capital letters. “mm” indicates the number of mismatches from the primary 17 bp *attB*. “occurrences” indicates the number of independent times an insertion in each site was identified. Percentages of the total (27) are indicated in parenthesis. The * next to *yqhG* indicates that two different ICE*Bs1* insertions were isolated in this gene, once in each orientation. **C.** Sequence logo of the ICE*Bs1* secondary attachment sites. Using Weblogo 3.3 [21], we generated a consensus motif of the 26 bases surrounding the insertion site of the 15 secondary insertion sites for ICE*Bs1*. For comparison, the primary attachment site for ICE*Bs1* is a 17 bp region with 5 bp inverted repeats and a 7 bp spacer region in the middle [10]. The size of each nucleotide corresponds to the frequency with which that nucleotide was observed in that position in the secondary attachment sites.

There were 15 different secondary integration sites for ICE*Bs1* among the 27 independent transconjugants (Figure 2). Seven of the 15 sites were described previously [10], and eight additional sites are reported here. There appears to be no absolute bias for the orientation of ICE*Bs1* insertions with respect to the direction of the replication forks, although 10 of the 15 insertions were oriented such that the direction of ICE*Bs1* replication was co-directional with the direction of the chromosomal replication forks (Figure 2A). Of the 27 independent transconjugants, 11 (41%) had ICE*Bs1* inserted in a site in *yrkM* (designated *yrkM*::ICE*Bs1*) (Figure 2B), a gene of unknown function. Three of the 27 (11%) transconjugants had ICE*Bs1* inserted in a site in *mmsA* (encoding an enzyme involved in myo-inositol catabolism [20]). The site in *yrkM* is the most similar to the primary attachment site *attB*, differing by two base pairs. The site in *mmsA* differs from *attB* by three base pairs (Figure 2B). Two insertions were in a site in *yqhG*, although in opposite orientations. These are counted as two different sites since the sequence in each orientation is different (Figure 2B). The remaining 11 insertions were in unique sites, either in genes or intergenic regions (Figure 2B). None of the identified insertions caused a noticeable defect in cell growth in rich (LB) or defined minimal medium when ICE*Bs1* was repressed. Furthermore, none of the insertions were in tRNA genes (including redundant, nonessential tRNA genes) that are common integration sites for many ICEs [8], [9].

Some of the secondary insertion sites were similar to and others quite different from the primary ICE*Bs1* attachment site (*attB*ICE*Bs1*, or simply *attB*). *attB* contains a 17 bp stem-loop sequence consisting of a 5 bp inverted repeat separated by 7 bp (Figure 2C). We aligned and compared the sequences of the 15 different secondary attachment sites and searched for a common motif using WebLogo 3.3 (http://weblogo.threeplusone.com/) [21]. For each secondary attachment site, we provided an input of 26 bp that included the region of the stem-loop sequence (17 bp, inferred from the sequence of *attB*) and a few base pairs upstream and downstream. The conserved sequences were largely in the 17 bp that were originally proposed to comprise *attB* [10], including several positions in the loop region of the stem-loop sequence, the 5 bp inverted repeats, and perhaps 1–2 additional base pairs downstream of the stem-loop (Figure 2C). There was considerable sequence diversity among the 15 secondary integration sites and the primary site *attB*, and no single position was conserved in all the secondary sites (Figure 2B). In some cases (e.g., insertions in *yrkM*, *mmsA*, *yqhG*, and *srfAA*) there are only 2–3 base pairs that are different between the secondary site and *attB*. In contrast, insertion sites in *yghL*, *yvbT*, and *ykrP* have 10–12 mismatches (out of 17 bp) from the sequence of *attB* (Figure 2B). These results indicate that in the absence of the primary integration site in *trnS-leu2*, ICE*Bs1* can integrate into many different sites throughout the genome, albeit at a lower efficiency [10]. Based on the diversity of the observed secondary attachment sites and the number of sites identified only once, it is clear that we have not identified all of the possible secondary integration sites for ICE*Bs1*.

### Integration into the secondary site in *ykrM* in the presence of a functional *attB*

We wondered if ICE*Bs1* could insert into a secondary site in cells in which the primary site, *attB*, is intact. To test this, ICE*Bs1* (from donor strain KM250) was transferred by conjugation to an ICE*Bs1*-cured recipient that contained *attB* (strain KM524). Transconjugants were selected on solid medium and ∼10^8^ independent transconjugants were pooled. DNA from the pooled transconjugants was then used as a template for quantitative real-time PCR (qPCR) with primers that detected ICE*Bs1* integrated into *yrkM* (the most frequently used secondary site). We found that the frequency of integration into *yrkM* was ∼10^−4^ to 10^−3^ of that into *attB*. As a control, we performed reconstruction experiments. Known amounts of DNA from two strains, one containing an insertion in *yrkM* (strain KM72), and the other containing an insertion in *attB* (strain AG174) were mixed and used as a template in qPCR, analogous to the experiment with DNA from the pooled transconjugants. These reconstruction experiments validated the results determined for the frequency of insertion into *yrkM*.

### Excision of ICE*Bs1* from secondary integration sites is reduced

**Figure 3:**
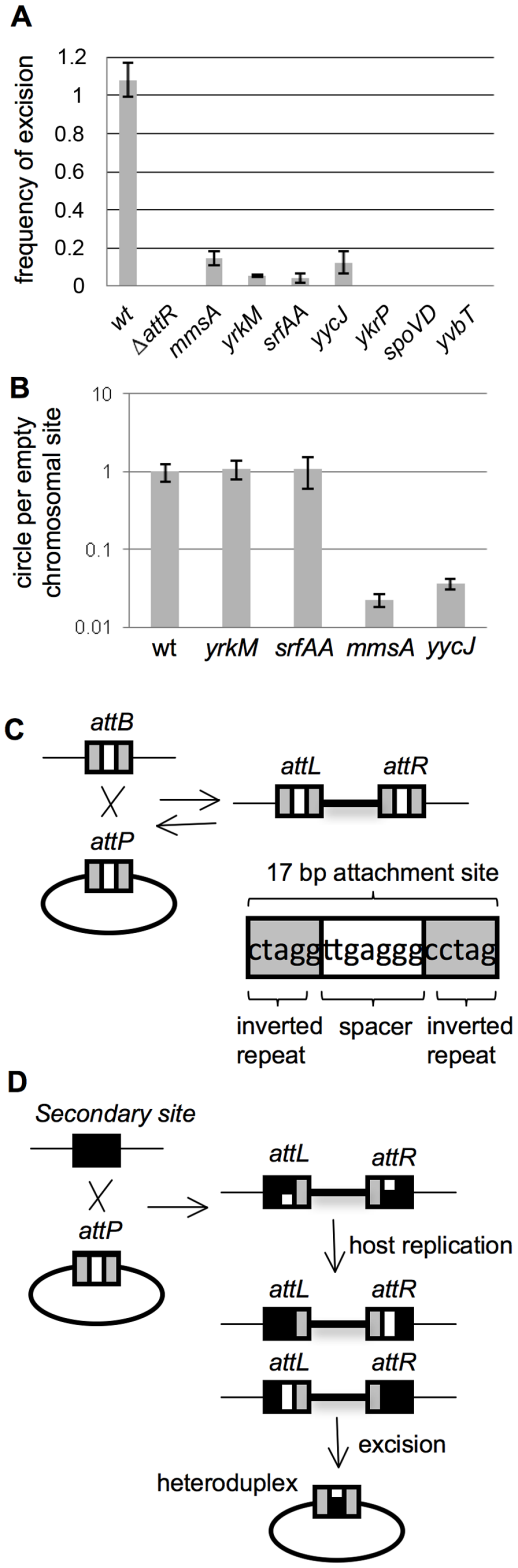
Excision of ICE*Bs1* from secondary attachment sites. **A–B.** Excision frequencies and relative amounts of the excision products (circular ICE*Bs1* and empty chromosomal site) were determined as described in Materials and Methods. Cells were grown in defined minimal medium with arabinose as carbon source. Products from excision were determined two hours after addition of xylose to induce expression of Pxyl-*rapI* to cause induction of ICE*Bs1* gene expression. Primers for qPCR were unique to each attachment site. Strains used include: wt, that is, ICE*Bs1* inserted in *attB* (CAL874); *ΔattR*, ICE*Bs1* integrated in *attB*, but with the right attachment site deleted and ICE*Bs1* unable to excise (Figure 1) (CAL872); *mmsA*::ICE*Bs1* (KM70); *yrkM*::ICE*Bs1* (KM72); *srfAA*::ICE*Bs1* (KM141); *yycJ*::ICE*Bs1* (KM132); *ykrP*::ICE*Bs1* (KM77); *spoVD*::ICE*Bs1* (KM130); *yvbT*::ICE*Bs1* (KM94). Each strain was assayed at least three times (biological replicates) and qPCR was done in triplicate on each sample. Error bars represent standard deviation. **A.** Frequency of excision of ICE*Bs1* from the indicated site of integration. The relative amount of the empty chromosomal attachment site was determined and normalized to the chromosomal gene *cotF*. Data were also normalized to a strain with no ICE*Bs1* (JMA222), which represents 100% excision. **B.** Relative amount of circular ICE*Bs1* compared to the amount of empty chromosomal attachment site for the indicated insertions. The relative amount of the ICE*Bs1* circle, normalized to *cotF*, was divided by the relative amount of the empty attachment site, also normalized to *cotF*. These ratios were then normalized to those for wild type. **C.** Cartoon of integration of ICE*Bs1* into its primary bacterial attachment site *attB*. *attB* is identical to the attachment site on ICE*Bs1*, *att*ICE*Bs1*. They consist of a 17 bp region with 5 bp inverted repeats (gray boxes) on each side of a 7 bp spacer region (white box). During integration and excision, a recombination event occurs in the 7 bp spacer (crossover) region [38]. **D.** Cartoon of integration of ICE*Bs1* into secondary integration sites. A secondary integration site is indicated with a black box. When ICE*Bs1* integrates into a secondary site, the crossover regions in *att*ICE*Bs1* and that of the secondary site are not necessarily identical, potentially creating a mismatch. This mismatch, if not repaired, will be resolved by host replication, generating left and right ends with different crossover sequences. Excision would then create a circular ICE*Bs1* with a heteroduplex in the attachment site on ICE*Bs1*.

We wished to determine if there were any deleterious consequences of integration of ICE*Bs1* into secondary attachment sites. We found that although ICE*Bs1* integrated into the secondary integration sites, excision from all of the secondary sites we analyzed was reduced or eliminated. We monitored excision from seven of the secondary sites by overexpressing the activator of ICE*Bs1* gene expression, RapI, from a regulated promoter (Pxyl-*rapI*) integrated in single copy in the chromosome at the nonessential gene *amyE* (Materials and Methods). Overproduction of RapI induces ICE*Bs1* gene expression [11], [22] and typically results in excision of ICE*Bs1* from *attB* in >90% of cells within 1–2 hrs [10], [15]. Following a similar protocol as described for monitoring excision from *attB* [11], [15], [22], we performed qPCR using genomic DNA as template and primers designed to detect the empty secondary attachment site that would form if the element excised. In a positive control, excision of ICE*Bs1* from *attB* occurred in >90% of cells within two hours after expression of the activator RapI (Figure 3A, wt). In a negative control, excision of an ICE*Bs1* Δ*attR* mutant (Figure 1C), integrated in *attB*, was undetectable (Figure 3A, Δ*attR*). Excision from four of the sites tested, *yrkM*, *mmsA*, *srfAA*, and *yycJ*, was reduced yet still detectable, ranging from 4% to 15% of that of ICE*Bs1* from *attB*. Excision from the other three sites tested, *yvbT*, *spoVD*, and *ykrP*, was undetectable (Figure 3A), similar to what we observed for ICE*Bs1* Δ*attR*, the excision-defective control. In general, the secondary integration sites that are most divergent from *attB* had the least amount of excision (Table 1).

**Table 1:** Summary of properties of several ICE*Bs1* insertions in secondary attachment sites.

Insertion site (#mm)^a^	Excision frequency^b^	Viability^c^	*dinC-lacZ* d
*attB*	1.0	1.0	1.0
*yrkM* (2)	0.06	0.030	34
*mmsA* (3)	0.15	0.14	6.1
*srfAA* (3)	0.04	0.10	8.7
*yycJ* (7)	0.12	0.073	N.D.
*spoVD* (8)	<10^−4^	0.010	N.D.
*ykrP* (12)	<10^−4^	0.040	4.1
*yvbT* (11)	<10^−4^	0.0038	24
Δ*attR*	<10^−4^	0.092	6.7

a site of insertion of ICE*Bs1*; #mm indicates the number of mismatches between the insertion site and *attB* (illustrated in Figure 2).

b excision frequency measured as the empty attachment site 2 hrs after induction of ICE*Bs1* gene expression; normalized to wt; same data as in Figure 3, except that here data is normalized to wt (*attB*). Excision frequency from *attB* was 1.

c cell viability normalized to ICE*Bs1* at *attB*; same data as in Figure 4. Viability of ICE*Bs1* at *attB* was 0.9 of uninduced.

d expression of damage inducible gene *dinC*-*lacZ*, normalized to that of cells with ICE*Bs1* in *attB*; data from Figure 6. ß-galactosidase specific activity of ICE*Bs1* at *attB* was 0.3.

These findings indicate that integration of ICE*Bs1* into sites other than *attB* causes a reduction, sometimes quite severe, in the ability of the element to excise. Because excision is required for transfer of a functional ICE, this reduced excision will limit the spread of ICE*Bs1* that has inserted into secondary sites from which it cannot escape.

### Decreased conjugation of ICE*Bs1* from secondary sites

We measured the mating efficiencies of ICE*Bs1* following excision from the four secondary attachment sites from which excision was reduced but detectable. Excision of ICE*Bs1* is required for transfer of the element to recipient cells. Thus, if the ICE*Bs1* circle is stable, then the mating efficiencies should be proportional to the excision frequency. The mating efficiencies of ICE*Bs1* from *yrkM* and *srfAA* were ∼2–5% of that of ICE*Bs1* from *attB*. Likewise, the excision frequencies of ICE*Bs1* inserted in *yrkM* and *srfAA* were ∼5% of those of ICE*Bs1* in *attB*. These results indicate that for ICE*Bs1* integrated in *yrkM* and *srfAA*, the mating efficiencies were approximately what was expected from the reduced excision frequencies.

In contrast, the mating efficiencies of ICE*Bs1* that excised from *mmsA* or *yycJ* were reduced beyond what would be expected from the already lowered excision frequency. In both cases, the excision frequencies were ∼15% of that of ICE*Bs1* integrated in *attB*. However, the mating efficiencies were ∼0.2% of that of ICE*Bs1* from *attB*, a 75-fold difference. Based on this result, we postulated that the reduced mating efficiency relative to the excision frequency was indicative of a reduction in the amount of circular ICE*Bs1*.

### Reduced levels of circular ICE*Bs1* from secondary sites that generate a heteroduplex

We measured the relative amounts of circular ICE*Bs1* after excision from *yrkM*, *srfAA*, *mmsA*, and *yycJ*, the four insertions with reduced but detectable excision, using qPCR primers designed to detect only the circular form of ICE*Bs1*. The relative amounts of each circle were compared to the relative amount of the empty secondary attachment site from which ICE*Bs1* excised. Measurements were made two hours after induction of ICE*Bs1* gene expression (overproduction of RapI).

As expected, the ratio of the amounts of the circular form to the empty attachment site was about the same for insertions in *yrkM* and *srfAA* as for an insertion in *attB* (Figure 3B). In contrast, the ratio of the circle to the empty attachment site for *mmsA* and *yycJ* was significantly less than that for wild type (Figure 3B). Comparing the total amount of the ICE*Bs1* circle from *mmsA* and *yycJ* to that from *attB* indicated that there was approximately 0.3% as much circle from each site as from *attB*. This decrease in the amount of ICE*Bs1* circle is consistent with and likely the cause of the drop in mating efficiency to approximately 0.2% of that of ICE*Bs1* from *attB*.

The decrease in the amount of circular ICE*Bs1* from *mmsA* and *yycJ* is likely due to the generation of a heteroduplex in the attachment site on the circular ICE*Bs1*. The ICE*Bs1* attachment site contains a 17 bp sequence with a 7 bp spacer region between 5 bp inverted repeats. Integrase-mediated site-specific recombination occurs in the 7 bp spacer (the crossover region) [10] (Figure 3C). If the 7 bp region in a chromosomal attachment site is different from that in ICE*Bs1*, as is the case for *mmsA* and *yycJ*, then integration and host replication will create left (*attL*) and right (*attR*) ends that have different crossover regions (Figure 3D). Upon excision, these elements are predicted to contain a heteroduplex in the attachment site on the excised circular ICE*Bs1*. Of the four insertions that have readily detectable excision frequencies, two (*mmsA* and *yycJ*) are predicted to form a heteroduplex and two (*yrkM* and *srfAA*) are not. In the case of *mmsA*::ICE*Bs1*, the left and right ends are known to have different sequences [10].

Together, our results indicate that excision of ICE*Bs1* from secondary sites from which a heteroduplex is formed leads to lower levels of the circular ICE*Bs1* heteroduplex and a reduction in the ability of ICE*Bs1* to transfer to other cells. We do not yet know what causes the lower amounts of the ICE*Bs1* heteroduplex. Loss of the DNA mismatch repair gene *mutS* did not alter the instability of the ICE*Bs1* heteroduplex (unpublished results), indicating that mismatch repair is not solely responsible for this effect. Nonetheless, the overall reduction in transfer is due to both decreased excision and further decreased amounts of the excised element. Both of these defects provide barriers to the spread of ICE*Bs1* from secondary attachment sites.

### ICE*Bs1* returns to *attB* following excision and conjugation from secondary sites

We found that when ICE*Bs1* excises from a secondary site and transfers to wild type cells via conjugation it tends to integrate in the primary attachment site, *attB*, and not in a secondary site. Donors with ICE*Bs1* in *yrkM*, *mmsA*, *yycJ*, and *srfAA* were crossed with a recipient (strain KM110) containing *attB* (and all known secondary sites). Individual transconjugants from each cross were isolated and tested by PCR for the presence of ICE*Bs1* in *attB*. ICE*Bs1* was present in *attB* in 9 of 10 transconjugants from *yrkM*::ICE*Bs1* donors, 9 of 9 transconjugants from *mmsA*::ICE*Bs1* donors, 9 of 10 transconjugants from *yycJ*::ICE*Bs1* donors, and 10 of 10 transconjugants from *srfAA*::ICE*Bs1* donors. In the two cases where ICE*Bs1* was not in *attB*, it was not present in the secondary site from which it came. We confirmed, using PCR primers internal to ICE*Bs1*, that ICE*Bs1* was present in the transconjugants. Thus, we conclude that even if ICE*Bs1* is able to excise from a secondary attachment site, there is a strong bias in returning to the primary site if that site is present in a transconjugant.

We also found that if *attB* is not present in recipients during conjugation, then ICE*Bs1* integrates into a secondary attachment site, but with no apparent bias for the site from which it originated. We crossed donors with ICE*Bs1* in *yrkM*, *mmsA*, and *srfAA* with a recipient missing *attB* (strain KM111), and tested individual transconjugants for integration into the cognate site from which ICE*Bs1* excised in the donor. With the *yrkM*::ICE*Bs1* donor, 1 of 6 transconjugants had ICE*Bs1* in *yrkM*. With the *mmsA*::ICE*Bs1* donor, none of the 10 transconjugants tested had ICE*Bs1* integrated in *mmsA*. With the *srfAA*::ICE*Bs1* donor, none of the four transconjugants tested had ICE*Bs1* in *srfAA*. Together, these results indicate that ICE*Bs1* has a strong preference to integrate into *attB*, even when it starts from a secondary site, and that if *attB* is not available, ICE*Bs1* tends to go to a secondary site, with no apparent preference for the original location.

### Decreased proliferation and viability of strains in which ICE*Bs1* has decreased excision

We found that strains with ICE*Bs1* in secondary integration sites had a decreased ability to form colonies when ICE*Bs1* gene expression was induced. We measured colony forming units (CFUs) of several strains with excision-defective (meaning reduced or no detectable excision) ICE*Bs1* insertions, including ICE*Bs1* in secondary sites and ICE*Bs1* Δ*attR* (in *attB*), both with and without induction of ICE*Bs1* gene expression. We also measured CFUs of wild type ICE*Bs1* (with normal excision frequencies) integrated at *attB* under similar conditions (Figure 4A). In the absence of RapI expression, when most ICE*Bs1* genes are repressed, growth and viability of excision-defective strains were indistinguishable from that of excision-competent strains. In contrast, by three hours after induction of ICE*Bs1* gene expression in excision-defective ICE*Bs1* strains (Δ*attR* with ICE*Bs1* in *attB*, or insertions in *mmsA*, *yrkM*, *srfAA*, *yycJ*, *spoVD*, *yvbT*, and *ykrP*), the number of CFUs was reduced compared to that of the excision-competent ICE*Bs1* (in *attB*) (Figure 4A). These results are consistent with previous observations that excision-defective *int* and *xis* null mutants have a viability defect when RapI is overproduced [10].

**Figure 4:**
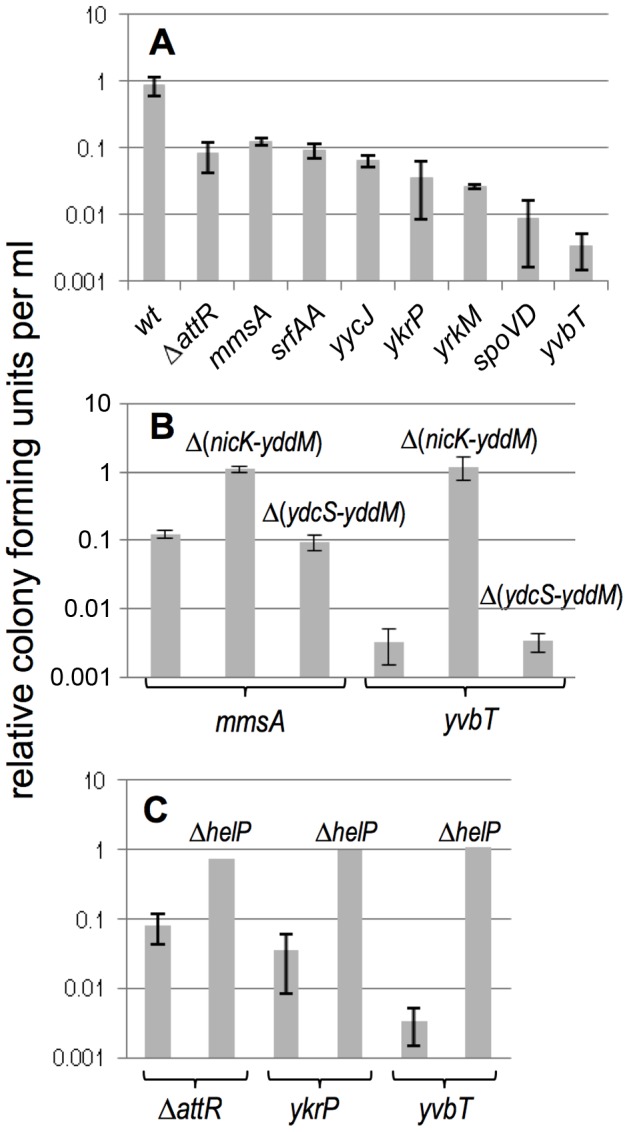
Effects of induction of ICE*Bs1* gene expression on cell viability. The effects of induction of ICE*Bs1* gene expression on cell viability are shown for the indicated insertions and their derivatives. Cells were grown in defined minimal medium with arabinose to early exponential phase (OD600∼0.05) and xylose was added to induce expression of Pxyl-*rapI*, causing induction of ICE*Bs1* gene expression. The number of colony forming units was measured three hours after induction and compared to cells grown in the absence of xylose (uninduced). All experiments were done at least three times, except for the *helP* mutants (panel C), which were done twice with similar results. Data presented are averages of the replicates. Error bars represent the standard deviation of at least three replicates. **A.** Drop in viability of strains in which excision of ICE*Bs1* is defective. Strains used include: wt, that is, *attB*::ICE*Bs1* (CAL874); *attB*::ICE*Bs1* Δ*attR*::*tet* (CAL872); *mmsA*::ICE*Bs1* (KM70); *srfAA*::ICE*Bs1* (KM141); *yycJ*::ICE*Bs1* (KM132); *ykrP*::ICE*Bs1* (KM77); *yrkM*::ICE*Bs1* (KM72); *spoVD*::ICE*Bs1* (KM130); *yvbT*::ICE*Bs1* (KM94). **B.** Data are shown for two secondary insertion sites (*mmsA*::ICE*Bs1* and *yvbT*::ICE*Bs1*). Similar results were obtained with *ykrP*::ICE*Bs1* and *srfAA*::ICE*Bs1* (data not shown). Derivatives of each insertion that delete *nicK* and all downstream ICE*Bs1* genes (Δ*nicK-yddM*) or that leave *nicK* intact and delete just the downstream genes (Δ*ydcS-yddM*) (Figure 1) were tested. Strains used include: *mmsA*::ICE*Bs1* (KM70); *mmsA*::{ICE*Bs1* Δ(*nicK-yddM*)::*cat*} (KM366); *mmsA*::{ICE*Bs1* Δ(*ydcS-yddM*)::*cat*} (KM358); *yvbT*::ICE*Bs1* (KM94); *yvbT*::{ICE*Bs1* Δ(*nicK-yddM*)::*cat*} (KM369); *yvbT*::{ICE*Bs1* Δ(*ydcS-yddM*)::*cat*} (KM362). Data for KM70 and KM94 are the same as those shown above in panel A and are shown here for comparison. **C.** The ICE*Bs1* helicase processivity protein encoded by *helP* is required for cell killing by ICE*Bs1*. Data are shown for two secondary integration sites (*ykrP* and *yvbT*) and the excision defective ICE*Bs1* Δ*attR*. The *helP* allele is a non-polar deletion [19]. Strains used include: *attB*::(ICE*Bs1* Δ*attR*::*tet*) (CAL872); *attB*::(ICE*Bs1* Δ*helP* Δ*attR*::*tet*) (KM437); *ykrP*::ICE*Bs1* (KM77); *ykrP*::(ICE*Bs1* Δ*helP*) (KM429); *yvbT*::ICE*Bs1* (KM94); *yvbT*::(ICE*Bs1* Δ*helP*) (KM459). Data for KM94, KM77, and CAL872 are the same as those shown above in panel A and are shown here for comparison.

Induction of ICE*Bs1* in several of the secondary integration sites (insertions in *mmsA*, *srfAA*, *yycJ* and ICE*Bs1* Δ*attR* in *attB*) caused a drop in CFU/ml to ∼10% of that of strains without ICE*Bs1* induction or the strain with wild type ICE*Bs1* at *attB* (Figure 4A). Induction of ICE*Bs1* in other insertion sites (*ykrP*, *yrkM*, *spoVD*, *yvbT*) caused a more severe drop in viability. The differences in CFU/ml between induced and uninduced cells (three hours after induction) appeared to be the combined effects of both a defect in proliferation (cell division) and cell death (viability). At times ≥3 hrs after induction of ICE*Bs1* gene expression, the number of CFU/ml dropped to below that before induction of gene expression, indicating that preexisting cells lost viability. For simplicity, we use “viability” to refer to both cell death and the decreased proliferation.

The drop in viability after induction of ICE*Bs1* in the various insertions did not correlate with dissimilarity of the attachment sites to *attB* or to the amount of residual excision in the excision-defective strains. For example, the ICE*Bs1* Δ*attR* mutant is completely unable to excise, and viability is ∼10% three hours after induction of ICE*Bs1* gene expression. In contrast, ICE*Bs1* inserted into *yrkM* has about 5% excision after induction of ICE*Bs1* gene expression and viability is ∼3% (Table 1). Together, these results indicate that something about the specific locations of the insertions is likely causing the more extreme viability defect observed in some of the excision-defective ICE*Bs1* strains.

One of the most extreme effects on viability after induction of ICE*Bs1* gene expression is from the insertion in *yvbT*. Within three hours after induction of ICE*Bs1* gene expression in the *yvbT*::ICE*Bs1* strain, viability was ∼0.3% of that of strains without ICE*Bs1* induction or of the strain with excision-competent ICE*Bs1* (Figure 4A). *yvbT* gene product is predicted to be similar to alkanal monooxygenases (luciferases). Insertion of ICE*Bs1* in *yvbT* likely knocks out *yvbT* function, so it seemed possible that the loss of *yvbT* combined with induction of ICE*Bs1* gene expression was causing the severe drop in viability. To test this hypothesis, we deleted *yvbT* in cells containing ICE*Bs1* inserted into *mmsA* and tested for viability after induction of ICE*Bs1* gene expression. There was no additional drop in viability of the *mmsA*::ICE*Bs1 yvbT* null mutant compared to the *mmsA*::ICE*Bs1* secondary site alone (wild type *yvbT*), either with or without induction of ICE*Bs1* gene expression. Based on these results, we conclude that the severe defect in viability of the *yvbT*::ICE*Bs1* secondary site mutant was not due to the loss of *yvbT* function combined with induction of ICE*Bs1* gene expression. It is also possible the severe drop in viability was due to production of a fragment of the *yvbT* gene product. This possibility seems highly unlikely because the putative fragment alone does not cause a phenotype, rather the drop in viability requires both induction and replication (see below) of ICE*Bs1*. In addition, other insertions also caused a severe drop in viability and it is highly unlikely that each one of these is producing a toxic protein fragment.

We do not know what causes the more severe drop in viability in some insertions. However, the decrease in cell proliferation and viability caused by expression of ICE*Bs1* in secondary attachment sites should provide selective pressure against the long term survival of these strains. The more severe the loss in viability, the stronger the selective pressure against long term survival of strains with insertions in these sites. Suppressor mutations that alleviate the drop in viability are readily obtained (KLM, C. Lee, ADG, data not shown), although most of these mutations have not been characterized.

### ICE*Bs1* replication functions are required for the drop in viability of excision-defective insertions

**Figure 5:**
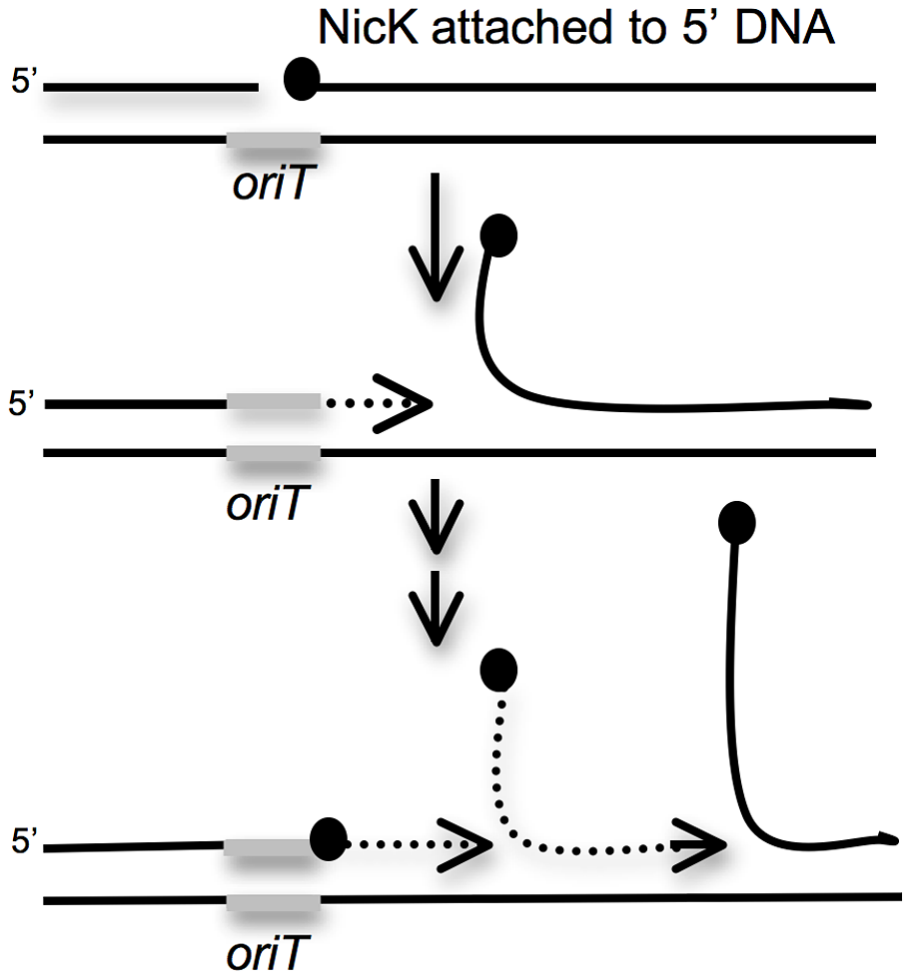
Cartoon of repeated rolling-circle replication from the ICE*Bs1 oriT* that is stuck in the chromosome. Rolling circle replication is induced in ICE*Bs1* insertions that are unable to excise from the chromosome. During this replication, the ICE*Bs1* relaxase NicK (black circles) nicks a site in *oriT*, the origin of transfer (gray bar) that also functions as an origin of replication [15], [23]. NicK presumably becomes covalently attached to the 5′ end of the nicked DNA. Replication extends (dotted line with arrow) from the free 3′-end, and regenerates a functional *oriT* that is a substrate for another molecule of NicK. The only other ICE*Bs1* product needed for ICE*Bs1* replication is the helicase processivity factor HelP [19]. The rest of the replication machinery (not shown) is composed of host-encoded proteins.

Because the drop in proliferation and viability in the first few hours after induction of ICE*Bs1* gene expression occurs in ICE*Bs1* excision-defective and not in excision-competent strains, the decreased viability is likely due to a cis-acting property of ICE*Bs1* and not a diffusible ICE*Bs1* product. One of the more dramatic changes following induction of ICE*Bs1* gene expression is induction of multiple rounds of unidirectional rolling circle replication [15]. This replication initiates from the ICE*Bs1* origin of transfer *oriT*, requires the ICE*Bs1* relaxase encoded by *nicK* and the helicase processivity factor encoded by *helP* (previously *ydcP*) [19]. Rolling circle replication of ICE*Bs1* occurs even when ICE*Bs1* is unable to excise from the chromosome as observed previously for a mutant unable to excise [15]. Therefore, we expected that induction of ICE*Bs1* gene expression in the secondary site insertions would lead to unidirectional rolling circle replication from *oriT* in the host chromosome (Figure 5). It seemed likely that this replication could cause damage to the chromosome and lead to the decrease in cell viability.

We tested *nicK* and the genes downstream for effects on cell viability following induction of ICE*Bs1* gene expression in the excision-defective insertions. Preliminary experiments indicated that loss of *nicK* restored viability after induction of ICE*Bs1* gene expression. However, this effect could have been due to polarity on downstream genes. Unfortunately, *nicK* null mutants are difficult to fully complement [23], perhaps because NicK might act preferentially in cis. In addition, complementation of other supposedly “non-polar” mutations in ICE*Bs1* are not complemented fully [19], [24]. Therefore, to test if loss of *nicK* was responsible for the suppression of lethality, or if the suppression was due to loss of expression of a downstream gene, we compared two different deletions in ICE*Bs1*. One deletion removed *nicK* and most of the downstream genes {Δ(*nicK-yddM*)} (Figure 1D). In the second deletion, *nicK* was intact, but most of the genes downstream from *oriT* and *nicK* were removed {Δ(*ydcS-yddM*)} (Figure 1E).

We found that deletion of *nicK* alleviated the growth defect of excision-defective secondary insertions, including *mmsA*::ICE*Bs1* and *yvbT*::ICE*Bs1* that caused the most severe drop in viability (Figure 4B). Deletion of the genes downstream from *nicK* did not alleviate the drop in viability (Figure 4B), indicating that expression of these genes (many encoding conjugation functions) was not the cause of the decreased cell viability. In addition, in preliminary experiments, we found that several suppressor mutations that restore viability to an excision-defective ICE*Bs1* (in this case, at *attB*) were null mutations in *nicK* (C. Lee, ADG, unpublished results). Together, these results indicate that a NicK-dependent process is causing the drop in viability of the excision-defective ICE*Bs1*.

NicK creates a nick at a specific site in ICE*Bs1 oriT* [23], and nicking is required for ICE*Bs1* replication (and conjugation) [15]. To determine if the drop in cell viability was due to nicking per se, or to replication, we used a recently defined ICE*Bs1* gene, *helP*, which encodes a helicase processivity factor that is needed for ICE*Bs1* replication but not for nicking [19], [23]. Deletion of *helP* (Figure 1F) is not polar on *nicK* and does not affect nicking at *oriT* [19]. Deletion of *helP* completely alleviated the growth defect associated with induction of ICE*Bs1* (Figure 4C).

Based on these results, we conclude that unidirectional rolling circle replication from *oriT* in the chromosome most likely caused the drop in viability of the excision-defective ICE*Bs1*. The decrease in viability could be due to breaks and degradation of chromosomal DNA around the site of insertion and/or disruptions in host chromosomal replication caused by the multiple rounds of rolling circle replication from *oriT* (Figure 5).

### Induction of the SOS response in strains in which ICE*Bs1* is defective in excision

**Figure 6:**
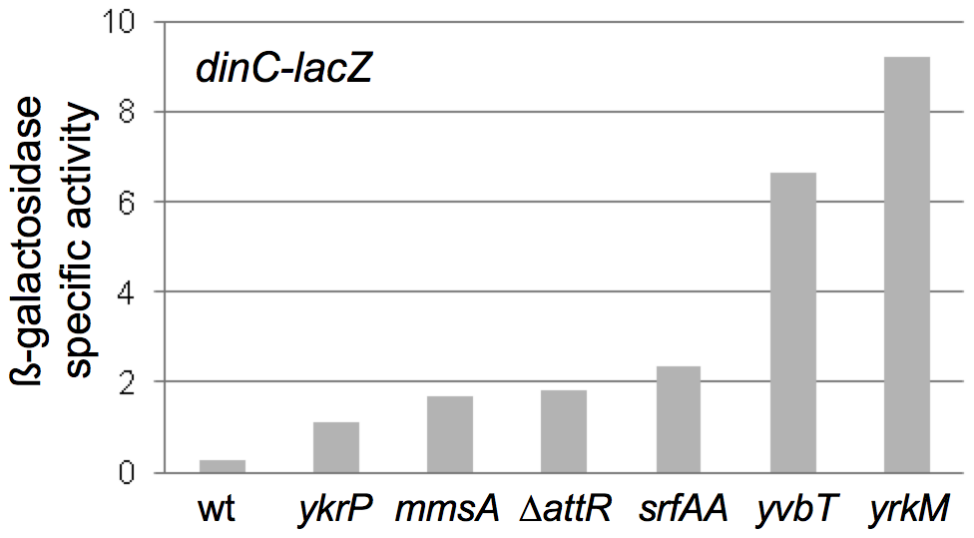
Induction of the SOS response. The ß-galactosidase specific activities from the SOS transcriptional reporter fusion *dinC*-*lacZ* in strains with ICE*Bs1* in the indicated secondary attachment sites are presented. Strains were grown as described in Figure 4 and samples for ß-galactosidase assays were taken 3 hours after induction of ICE*Bs1* gene expression. Data presented are the averages of two biological replicates (four for Δ*attR* strain KM392)). For all of the strains with insertions in secondary attachment sites, the values from the biological replicates were within 20% of the average. Strains used include: wt, *attB*::ICE*Bs1* (KM390); *ykrP*::ICE*Bs1* (KM402); *mmsA*::ICE*Bs1* (KM394); *attB*::ICE*Bs1* Δ*attR*::*tet* (KM392); *srfAA*::ICE*Bs1* (KM400); *yvbT*::ICE*Bs1* (KM396); *yrkM*::ICE*Bs1* (KM404).

We found that induction of ICE*Bs1* gene expression in the excision-defective insertions caused induction of the host SOS response. Like that in other organisms, the SOS response in *B. subtilis* results in increased expression of a large set of genes in response to DNA damage or replication stress [25]. We used a *lacZ* fusion to a damage-inducible gene, *dinC-lacZ* [26], [27], to monitor the SOS response in cells following induction of ICE*Bs1*. Without induction of ICE*Bs1* gene expression, there was no detectable ß-galactosidase activity above background levels, indicating that none of the insertions alone caused elevated SOS gene expression. In all of the excision-defective ICE*Bs1* strains analyzed (ICE*Bs1* Δ*attR* in *attB*, and insertions in *mmsA*, *yvbT*, *ykrP*, *srfAA*, and *yrkM*), there was a ≥3.5-fold increase in ß-galactosidase levels from the *dinC-lacZ* fusion 3 hrs after induction of ICE*Bs1* gene expression (Figure 6). In contrast, there was no detectable increase in ß-galactosidase activity three hrs after induction of ICE*Bs1* gene expression in the excision-competent insertion in *attB* (Figure 6). There was no apparent correlation between the amount of SOS induction and the severity of the viability defect. For example, one of the strains with the most severe viability defect (ICE*Bs1* in *ydcP*) had a relatively low amount of expression of *dinC*-*lacZ* (Figure 6). However, the amount of SOS induction could be an underestimate since many cells in the population lose viability.

Induction of *dinC-lacZ* in the strains with ICE*Bs1* in secondary attachment sites was consistent with prior preliminary experiments using DNA microarrays that indicated induction of the SOS response in ICE*Bs1 int* and *xis* mutants that are incapable of excision (N. Kavanaugh, C. Lee, ADG, unpublished results). Based on these results, we conclude that induction of ICE*Bs1* gene expression in cells in which ICE*Bs1* is stuck in the chromosome causes DNA damage that induces the host SOS response. However, the SOS response per se is not what caused cell death.

## Discussion

We isolated and characterized insertions of the integrative and conjugative element ICE*Bs1* of *B. subtilis* into secondary integration (attachment or insertion) sites. Secondary integration sites appear to be used naturally, even in the presence of the primary site, at a frequency of ∼10^−4^ to 10^−3^ of that of the primary site, indicating that approximately 100–1,000 cells in a population of ∼10^6^ transconjugants will have ICE*Bs1* at a secondary site. We found that insertions in secondary sites are detrimental for the propagation of ICE*Bs1* and detrimental to the survival of the host cells. These detrimental effects likely provide selective pressure to maintain the already established site-specificity. Below we discuss target site selection among ICEs, aspects of ICE*Bs1* biology that make insertions into secondary sites detrimental, and the more general implications for the evolution of ICEs.

### Target site selection and maintenance of tRNA genes as integration sites

We have identified 15 different secondary insertion sites for ICE*Bs1*. Some of these sites are similar to the primary attachment site, but some are quite different. Based on the diversity of sites, and the isolation of only a single insertion in many of them, it is likely that we are nowhere near saturation for identifying all possible sites in non-essential regions. Given that there is some sequence conservation among the secondary sites, DNA sequence is clearly important in the potential function as an integration site. However, we suspect that other factors also contribute. These factors could include possible roles for nucleoid binding proteins, other DNA binding proteins, transcription, and local supercoiling.

Many site-specific ICEs have preferred integration sites in tRNA genes. This preference is thought to occur, at least in part, because tRNA genes are highly conserved and contain inverted repeats that are typically used as integration targets for site-specific recombinases [9]. We postulate that the selective pressure to maintain site-specific integration in a tRNA gene comes from a combination of factors, including: the conservation of tRNAs, the ability of an ICE to efficiently excise from the primary attachment site, and the decreased cell viability and decreased ability of an ICE to spread when excision is reduced due to integration into a secondary site.

### Selective pressures against ICEs in secondary attachment sites

Our results indicate that there are at least two main types of selective pressures against propagation of ICE*Bs1* that has inserted into a secondary integration site. First, there is strong pressure against the spread of that particular element due to the large defect in its ability to excise and the instability of circular ICE*Bs1* when it forms a heteroduplex. The excised circular form of an ICE is necessary for its complete transfer to a recipient cell. At least one other ICE has a reduced excision frequency from a secondary integration site. Excision of SXT from a secondary attachment site in *Vibrio cholerae* was reduced 3–4-fold relative to its ability to excise from the primary attachment site [28]. In addition, lysogenic phages can also have reduced excision efficiencies from secondary attachment sites [29]. Insertion of any type of mobile genetic element into a location from which it has trouble getting out will be deleterious to the further horizontal propagation of that element. Based on our results, this is particularly true for ICE*Bs1*.

In addition to the defect in ICE*Bs1* excision and transfer from secondary integration sites, there is a decrease in cell viability following induction of ICE*Bs1* gene expression. ICE*Bs1* gene expression is normally induced under conditions of starvation or cell crowding when the activator RapI is expressed and active, or when the RecA-dependent SOS response is induced [22]. Induction of ICE*Bs1* gene expression causes rolling circle replication from the ICE*Bs1* origin of transfer *oriT* [15], [19]. Our results indicate that rolling circle replication from an element that is unable to efficiently excise from the chromosome causes a drop in cell viability. This drop is likely due to chromosomal damage and stalling of the chromosomal replication forks when they reach the complex structure formed by repeated initiation of rolling circle replication from *oriT* in the chromosome (Figure 5).

We suspect that autonomous replication is a common property of many ICEs but has not been generally observed because of the low frequency of induction and excision of most of these elements. There are indications that some other ICEs undergo autonomous replication [1], [16]–[18]. If autonomous replication of ICEs is widespread, as we postulate [15], [19], then there should be selective pressure against viability of cells in which an ICE is induced, replicates, and is unable to excise.

There were at least two different effects caused by replication of excision-defective elements. Replication from ICE*Bs1* in the chromosome caused a drop in cell viability of at least 10-fold, but sometimes caused a severe drop, 100–1000-fold in about 3 hrs. We do not know what causes this severe drop in viability, but it requires active replication of the ICE*Bs1* that is unable to excise from a specific chromosomal location. This severe drop in viability could be due to increased dosage of nearby genes or perhaps differential fragility of these chromosomal regions. In any case, the severe drop in viability provides even stronger selective pressure against propagation of the strains with insertions of ICE*Bs1* in these locations.

The growth defect associated with the secondary insertions is most obvious when ICE*Bs1* gene expression is induced. Cells with ICE*Bs1* insertions in secondary attachment sites might be purged from the population under natural conditions of induction, providing selective pressure against maintenance of integrants in secondary sites and favoring a site-specific strategy of integration and excision.

We estimated the effects of insertions in secondary sites in populations without experimentally induced activation of ICE*Bs1*. The “spontaneous” activation and excision frequency of ICE*Bs1* in a population of cells is estimated to be approximately one cell in 10^4^–10^5^ [10], [22], [30]. Assuming a frequency of activation of ICE*Bs1* of ∼10^−4^ per generation, and that all activated cells with ICE*Bs1* in a secondary site die, we estimate that it would take ∼23,000 generations for a population of cells with ICE*Bs1* in a secondary site to be 0.1 times the size of a population of cells with ICE*Bs1* in the primary site. The activation frequency increases under several conditions likely to be more relevant than growth in the lab, including: the presence of cells without ICE*Bs1*, entry into stationary phase, and during the SOS response [10], [11], [22]. For example, if activation of ICE*Bs1* actually occurs in 0.1% of cells, then it would take ∼2,300 generations for the secondary site insertion population to be 0.1 times the population of cells with ICE*Bs1* in the primary site. These effects are difficult to measure experimentally, but easy to see when ICE*Bs1* is efficiently induced.

### ICEs with single versus multiple integration sites

ICEs of the Tn*916*/Tn*1545* family can integrate into multiple sites in many organisms, yet they are not known to cause a defect in cell growth when gene expression is induced. Tn*916* and most family members contain *tetM*, a gene encoding resistance to tetracycline. Expression of *tetM* and Tn*916* genes is induced in the presence of tetracycline [31]. Tn*916* has two *helP* (helicase processivity) homologues and we predict that it undergoes autonomous rolling circle replication [19]. Despite relatively low excision frequencies, tetracycline-induced Tn*916* gene expression is not known to cause a drop in cell viability. Tetracycline induces expression of several Tn*916* genes, including those needed for excision. However, the Tn*916* relaxase (*orf20*), the two *helP* homologues (*orf22* and *orf23*), and the conjugation genes are not expressed until Tn*916* excises and circularizes [31]. Based on analogy to ICE*Bs1*, we have postulated that Tn*916* is capable of autonomous rolling circle replication [15] and that the relaxase (*orf20*) and at least one of the *helP* homologues are likely needed for this replication [19]. The regulation of Tn*916* gene expression specifically prevents expression of these putative replication functions until after excision. Consequently, rolling circle replication of Tn*916* cannot occur while the element is integrated in the chromosome. We speculate that some of the evolutionary pressures to establish and maintain a high degree of site specificity is lost when expression of ICE replication functions does not occur until after excision from the host genome.

## Materials and Methods

### Media and growth conditions

*Bacillus subtilis* was grown at 37°C in LB or defined S7_50_ minimal medium with arabinose (1%) as carbon source. Antibiotics and other chemicals were used at the following concentrations: isopropyl β-D-1-thiogalactopyranoside (IPTG) (1 mM), chloramphenicol (*cat*, 5 µg/ml), kanamycin (*kan*, 5 µg/ml), spectinomycin (*spc*, 100 µg/ml), erythromycin (0.5 µg/ml) and lincomycin (12.5 µg/ml) together, to select for macrolide-lincosamide-streptogramin B resistance (*mls* or *erm*).

### *Bacillus subtilis* strains and alleles

*B. subtilis* strains used are listed in Table 2. All except BTS14 are derived from AG174 (JH642) and contain mutations in *trpC* and *pheA* (not shown). Most of the strains were constructed using natural transformation or conjugation, as described below. Many alleles were previously described. *dinC18*::Tn*917lac* is an insertion in the damage-inducible gene *dinC* and creates a transcriptional fusion to *lacZ* [27]. Most ICE*Bs1* strains contained a kanamycin-resistance cassette {Δ(*rapI-phrI*)*342*::*kan*} [11]. ICE*Bs1* was induced by overexpression of *rapI* from a xylose-inducible promoter using *amyE*::{(Pxyl-*rapI*), *spc*} [24] or from an IPTG-inducible promoter using *amyE*::{(Pspank(hy)-*rapI*), *spc*} [11]. Δ*attR100*::*tet* deletes 216 bp spanning the junction between the right end of ICE*Bs1* and the chromosome [10]. Δ*helP155* is an unmarked 413-bp deletion that removes the entire coding sequence and the 35 bp *helP-ydcQ* intergenic region (Figure 1F) [19].

**Table 2:** *B. subtilis* strains used.

Strain	relevant genotype (comment and/or reference)
AG174	*phe trp* [39]
AG1624	*zbj-82*::Tn*917* (insertion at 65°) [40]
BTS13	PY79 (*trp* ^+^ *phe* ^+^) Δ*mutSL*::*spc* [41]
CAL522	*trnS-leu1-522* Δ*attB*::*cat*
CAL572	*yomR572*::(ICE*Bs1* Δ(*rapI-phrI*)*342*::*kan*) Δ*attB*::*cat comK*::*cat*::*spc* [10]
CAL575	*yvbT575*::(ICE*Bs1* Δ(*rapI-phrI*)*342*::*kan*) Δ*attB*::*cat comK*::*cat*::*spc* [10]
CAL576	*yqhG576*::(ICE*Bs1* Δ(*rapI-phrI*)*342*::*kan*) Δ*attB*::*cat comK*::*cat*::*spc* [10]
CAL577	*yobJ577*::(ICE*Bs1* Δ(*rapI-phrI*)*342*::*kan*) Δ*attB*::*cat comK*::*cat*::*spc* [10]
CAL578	Intergenic *ygxA rrnD-16S-578*::(ICE*Bs1* Δ(*rapI-phrI*)*342*::*kan*) Δ*attB*::*cat comK*::*cat*::*spc* [10]
CAL872	Δ*attR100*::*tet Δ*(*rapI-phrI*)*342*::*kan amyE*::{(Pxyl*-rapI*) *spc*}
CAL874	Δ(*rapI-phrI*)*342*::*kan amyE*::{(Pxyl*-rapI*) *spc*} [15]
JMA168	Δ(*rapI-phrI*)*342*::*kan amyE*::{(*Pspank*(*hy*)*-rapI*) *spc*} [23]
J3	*srfAA3*::(ICE*Bs1* Δ(*rapI-phrI*)*342*::*kan*) *trnS-leu1-522* Δ*attB*::*cat*
J4	*yycJ4*::(ICE*Bs1* Δ(*rapI-phrI*)*342*::*kan*) *trnS-leu1-522* Δ*attB*::*cat*
J9	*yrkM9*::(ICE*Bs1* Δ(*rapI-phrI*)*342*::*kan*) *trnS-leu1-522* Δ*attB*::*cat*
J11	*yqhG11*::(ICE*Bs1* Δ(*rapI-phrI*)*342*::*kan*) *trnS-leu1-522* Δ*attB*::*cat*
J12	*yisQ12*::(ICE*Bs1* Δ(*rapI-phrI*)*342*::*kan*) *trnS-leu1-522* Δ*attB*::*cat*
J14	*mmsA14*::(ICE*Bs1* Δ(*rapI-phrI*)*342*::*kan*) *trnS-leu1-522* Δ*attB*::*cat*
J16	*ykrP16*::(ICE*Bs1* Δ(*rapI-phrI*)*342*::*kan*) *trnS-leu1-522* Δ*attB*::*cat*
JMA222	ICE*Bs1* ^0^/cured of ICE*Bs1* [11]
KI1254	*dinC18*::Tn*917lac*; allele originally from YB5018 [27]
KM5	*yghL5*::(ICE*Bs1* Δ(*rapI-phrI*)*342*::*kan*) *trnS-leu1-522* Δ*attB*::*cat*
KM8	*spoVD8*::(ICE*Bs1* Δ(*rapI-phrI*)*342*::*kan*) *trnS-leu1-522* Δ*attB*::*cat*
KM10	*ydbJ10*::(ICE*Bs1* Δ(*rapI-phrI*)*342*::*kan*) *trnS-leu1-522* Δ*attB*::*cat*
KM70	*mmsA15*::(ICE*Bs1* Δ(*rapI-phrI*)*342*::*kan*) *amyE*::{(Pxyl*-rapI*) *spc*}
KM72	*yrkM9*(*J9*)::(ICE*Bs1* Δ(*rapI-phrI*)*342*::*kan*) *amyE*::{(Pxyl*-rapI*) *spc*}
KM77	*ykrP16*::(ICE*Bs1* Δ(*rapI-phrI*)*342*::*kan*) *amyE*::{(Pxyl*-rapI*) *spc*}
KM94	*yvbT575*::(ICE*Bs1* Δ(*rapI-phrI*)*342*::*kan*) *amyE*::{(Pxyl*-rapI*) *spc*}
KM110	ICE*Bs1* ^0^ *zbj*-82::Tn*917* (insertion at 65°)
KM111	ICE*Bs1* ^0^ *zbj*-82::Tn*917 trnS-leu1-522* Δ*attB*::*cat*
KM130	*spoVD8*::(ICE*Bs1* Δ(*rapI-phrI*)*342*::*kan*) *amyE*::{(Pxyl*-rapI*) *spc*}
KM132	*yycJ4*::(ICE*Bs1* Δ(*rapI-phrI*)*342*::*kan*) *amyE*::{(Pxyl*-rapI*) *spc*}
KM141	*srfAA3*::(ICE*Bs1* Δ(*rapI-phrI*)*342*::*kan*) *amyE*::{(Pxyl*-rapI*) *spc*}
KM250	ICE*Bs1* Δ(*rapI-phrI*)*342*::*kan amyE*::{(Pxyl*-rapI*) *cat*}
KM252	*mmsA15*::(ICE*Bs1* Δ(*rapI-phrI*)*342*::*kan*) *amyE*::{(Pxyl*-rapI*) *cat*}
KM268	*mmsA15*::(ICE*Bs1* Δ(*rapI-phrI*)*342*::*kan*) *amyE*::{(Pxyl*-rapI*) *cat*} Δ*mutSL*::*spc*
KM304	*mmsA15*::(ICE*Bs1* Δ(*rapI-phrI*)*342*::*kan*) *amyE*::{(Pxyl*-rapI*) *spc*} Δ*yvbT*::*cat*
KM358	*mmsA15*::(ICE*Bs1* Δ(*ydcS-yddM*)*356*::*cat*) *amyE*::{(Pxyl*-rapI*) *spc*}
KM362	*yvbT575*::(ICE*Bs1* Δ(*ydcS-yddM*)*356*::*cat*) *amyE*::{(Pxyl*-rapI*) *spc*}
KM366	*mmsA15*::(ICE*Bs1* Δ(*nicK-yddM*)*354*::*cat*) *amyE*::{(Pxyl*-rapI*) *spc*}
KM369	*yvbT575*::(ICE*Bs1* Δ(*nicK-yddM*)*354*::*cat*) *amyE*::{(Pxyl*-rapI*) *spc*}
KM384	*srfAA3*::(ICE*Bs1* Δ(*ydcS-yddM*)*356*::*cat*) *amyE*::{(Pxyl*-rapI*) *spc*}
KM386	*srfAA3*::(ICE*Bs1* Δ(*nicK-yddM*)*354*::*cat*) *amyE*::{(Pxyl*-rapI*) *spc*}
KM388	*ykrP16*::(ICE*Bs1* Δ(*ydcS-yddM*)*356*::*cat*) *amyE*::{(Pxyl*-rapI*) *spc*}
KM389	*ykrP16*::(ICE*Bs1* Δ(*nicK-yddM*)*354*::*cat*) *amyE*::{(Pxyl*-rapI*) *spc*}
KM390	Δ(*rapI-phrI*)*342*::*kan amyE*::{(Pxyl*-rapI*) *spc*} *dinC18*::Tn*917*(*lacZ mls*)
KM392	Δ*attR100*::*tet Δ*(*rapI-phrI*)*342*::*kan amyE*::{(Pxyl*-rapI*) *spc*} *dinC18*::Tn*917lac*
KM394	*mmsA15*::(ICE*Bs1* Δ(*rapI-phrI*)*342*::*kan*) *amyE*::{(Pxyl*-rapI*) *spc*} *dinC18*::Tn*917lac*
KM396	*yvbT575*::(ICE*Bs1* Δ(*rapI-phrI*)*342*::*kan*) *amyE*::{(Pxyl*-rapI*) *spc*} *dinC18*::Tn*917lac*
KM400	*srfAA3*::(ICE*Bs1* Δ(*rapI-phrI*)*342*::*kan*) *amyE*::{(Pxyl*-rapI*) *spc*} *dinC18*::Tn*917lac*
KM402	*ykrP16*::(ICE*Bs1* Δ(*rapI-phrI*)*342*::*kan*) *amyE*::{(Pxyl*-rapI*) *spc*} *dinC18*::Tn*917lac*
KM404	*yrkM9*(*J9*)::(ICE*Bs1* Δ(*rapI-phrI*)*342*::*kan*) *amyE*::{(Pxyl*-rapI*) *spc*} *dinC18*::Tn*917lac*
KM429	*ykrP16*::(ICE*Bs1* Δ*helP Δ*(*rapI-phrI*)*342*::*kan*) *amyE*::{(Pxyl*-rapI*) *spc*}
KM437	Δ*attR100*::*tet* Δ*helP Δ*(*rapI-phrI*)*342*::*kan amyE*::{(Pxyl*-rapI*) *spc*}
KM524	ICE*Bs1* ^0^ (*attB* ^+^) *amyE*::(*lacZ*, *mls*); used as recipient in conjugation to detect insertions in secondary sites
KM459	*yvbT575*::(ICE*Bs1* Δ*helP Δ*(*rapI-phrI*)*342*::*kan*) *amyE*::{(Pxyl*-rapI*) *spc*}
MMB868	*amyE*::{(Pxyl*-rapI*) *cat*}
MMB869	*amyE*::{(Pxyl*-rapI*) *spc*} [30]
REM54	Δ*attB*::*cat* [10]

#### Δ*attB* mutant with a compensatory mutation in *trnS*-*leu1*

Δ*attB*::*cat* is a deletion-insertion that is missing ICE*Bs1* and removes 185 bp that normally contains the primary chromosomal ICE*Bs1* attachment site, resulting in the loss of a functional *trnS-leu2* [10]. Although *trnS-leu2* is non-essential [10], [32], cells with Δ*attB* do not grow as well as wild type. To improve the growth of Δ*attB*::*cat*, we used a compensatory mutation in *trnS-leu1* that changes the anti-codon to that normally found in *trnS-leu2* (C. Lee, & ADG), analogous to the *leuF1* mutation previously described [32]. The compensatory mutation was constructed by site-directed mutagenesis using the overlap-extension PCR method [33]. Because *trnS-leu1* and Δ*attB*::*cat* are genetically linked, we selected for chloramphenicol resistant colonies and screened for the single bp mutation in *trnS-leu1* by sequencing. In addition to the mutant *trnS-leu1* allele (*trnS-leu1-522*), the strain had an additional mutation, (5′-CAAAAAAACTAA**A** to 5′-CAAAAAAACTAA**G** ) in the non-coding region between Δ*attB*::*cat* and *yddN*. Growth of the resulting strain, CAL522, was indistinguishable from that of wild type. This strain stably acquired ICE*Bs1* in conjugation experiments at a frequency ∼0.5% of that of wild type, approximately 10-fold lower than the strain without the compensatory mutation in *trnS-leu1* [10]. We do not understand the cause of this reproducible difference.

#### Deletion of *nicK* and downstream genes

We constructed two large deletion-insertion mutations in ICE*Bs1*, one removing *nicK* and all downstream genes, Δ(*nicK-yddM*)::*cat*, and the other leaving *nicK* intact, but removing the downstream genes, Δ(*ydcS-yddM*)::*cat*. Both deletions leave the ends of ICE*Bs1* intact (Figure 1D, E), have *cat* (chloramphenicol resistance) from pGEMcat [34], and were constructed using long-flanking homology PCR [35]. The Δ(*nicK-yddM*)::*cat* allele contains the first 127 bp in the 5′ end of *nicK*. The Δ(*ydcS-yddM*)::*cat* allele contains the first 29 bp in the 5′ end of *ydcS*. Both deletions (Figure 1) extend through the first 170 bp in *yddM*. The alleles were first transformed into wild type strain AG174. Chromosomal DNA was then used to transfer the alleles into other strains, including KM70 (*mmsA*::ICE*Bs1*), KM94 (*yvbT*::ICE*Bs1*), KM77 (*ykrP*::ICE*Bs1*), KM141 (*srfAA*::ICE*Bs1*), and CAL874 (ICE*Bs1* at *attB*). In all cases, the incoming deletion associated with *cat* replaced the Δ(*rapI-phrI*)*342*::*kan* allele present in ICE*Bs1* in the recipient.

#### Deletion of *yvbT* in *mmsA*::ICE*Bs1*

We constructed a deletion-insertion that removes the 19 bases before *yvbT* and the first 808 bp of *yvbT*, leaving the last 200 bp intact. The sequence from *yvbT* was replaced with *cat*, from pGEMcat [34], using long-flanking homology PCR [35]. The insertion-deletion was verified by PCR and the mutation was introduced into strain KM70 (*mmsA*::ICE*Bs1*) by transformation.

### Isolation and identification of secondary ICE*Bs1* integration sites

#### Mating ICE*Bs1* into a Δ*attB* recipient

Mating assays were performed essentially as described [10], [11]. Excision of a kanamycin resistant ICE*Bs1* (ICE*Bs1* Δ(*rapI-phrI*)*342*::*kan*) was induced in the donor cells by overproduction of RapI from Pspank(hy)-*rapI*. Donors (resistant to kanamycin and spectinomycin) were mixed with an approximately equal number of recipients (resistant to chloramphenicol) and filtered on sterile cellulose nitrate membrane filters (0.2 µm pore size). Filters were cut into 8 pieces (so that transconjugants were independent isolates), placed on Petri plates containing LB and 1.5% agar, and incubated at 37°C for 3 hours. Cells from each piece of filter were streaked for independent transconjugants by selecting for the antibiotic resistance conferred by the incoming ICE*Bs1* (kanamycin) and the resistance unique to the recipient (chloramphenicol). The recipient used in this report {Δ*attB*::*cat trnS-leu1-522*} is different from the recipient {Δ*attB*::*cat*} used previously [10]. The *trnS-leu1-522* confers normal growth to the Δ*attB* (Δ*trnS-leu2*) mutant (see above).

#### Inverse PCR to identify the site of insertion of independent transonjugants

Identification of integration sites was done essentially as described previously [10]. Briefly, we used inverse PCR to amplify the junction between the chromosome and the right (*yddM*) end of ICE*Bs1* integrated into various secondary sites. Chromosomal DNA was digested with HindIII and approximately 50 ng was ligated in a 100 µl reaction to favor circularization of DNA fragments. One-fourth of the ligation reaction was used in inverse PCR with either of two primer pairs (CLO17-CLO58 or CLO50-oJMA97) designed to amplify the ICE*Bs1* and chromosomal sequences flanking *yddM*. PCR products were sequenced with primers CLO17, CLO50, oJMA207, and CLO114 (primers are described in Table 3). Comparison to the *B. subtilis* genome sequence indicated where ICE*Bs1* had integrated.

**Table 3:** Primers used.

Name	Sequence^1^	Location, use, reference^2^
ABO14	CCAACGCAAAGATACCTTGC	5′ *yvbT*; qPCR
ABO15	TGTTCAGCAAGCCAGTAACG	3′ *yvbT*; qPCR
ABO17	CTGACATATACCACGCCCAC	5′ *yrkM*; qPCR
ABO18	AAACGCAATCCGCTACTTCC	5′ *mmsA*; qPCR
ABO19	GTATCATTGATGCGGCCCAG	near left end of ICE*Bs1*, 3′ in *trnS-leu2*; qPCR to detect ICE*Bs1* circle or left junction in chromosome
CLO109	GATATCTTGCCGTCACCACT	3′ *mmsA*; qPCR
CLO114	CTTAATGCTATAAATAAAGGCTTTTG	in ICE*Bs1*, near and extending towards the right end, in same direction as transcription of *trnS-leu2*; PCR, qPCR, sequencing
CLO116	CGCAGAGAGTTGCTGGTAAC	just upstream of *yrkM* (35 codons), in same direction as transcription of *yrkM* (5′); qPCR, PCR
CLO117	TGTAGAGTTCCTTGGCCTCT	just downstream of *yrkM*, in opposite direction of transcription of *yrkM* (3′); qPCR, PCR
CLO17	CCATTTACTGCCCAGAATAAATAACAAATCATG	in ICE*Bs1*, near and extending towards the right end, in same direction as transcription of *trnS-leu2* [10]; ∼50 bp farther from the right end of ICE*Bs1* than CLO114; PCR, qPCR, sequencing
CLO257	GGATGAACGCAGAACATTGG	5′ *cotF*, chromosomal gene for reference; qPCR
CLO258	GCTCAACACCCTGAATAGAC	3′ *cotF*, chromosomal gene for reference; qPCR
CLO261	GCCTACTAAACCAGCACAAC	5′, just upstream of *trnS-leu2*; qPCR [15], [30]
CLO262	AGCAAGTCTTCTCCCATAGC	3′, just outside the right end of ICE*Bs1*; qPCR [15], [30]
CLO264	TATTGAGATGCGGCCGAGAG	3′ *trnS-leu2*, downstream from *attB*; qPCR, to detect integration of ICE*Bs1* in *attB*
CLO273	AGGGCGAACTATGAGTTTGC	near and extending towards right end of ICE*Bs1*;qPCR, to detect integration of ICE*Bs1* in *attB*
CLO50	GCCTTCTGCGTCCGGTCG	In *kan*; near right end of ICE*Bs1* Δ(*rapI*-*phrI*)::*kan*; inverse PCR, sequencing [10]
CLO58	CGCGGATCCGACTGTACCGTACGTTTTTAAAGATGATGTAAC	in *yddM*; inverse PCR, sequencing [10]
KM15	AGTCCGCTTACCAGGGTAAC	qPCR; 3′ *ykrP*; qPCR
KM16	GAGCTTGTCACGGACATTCG	qPCR; 5′ *ykrP*; qPCR
KM18	TATACAGCCAAAGCGGAGTG	qPCR; 3′ *yycJ*; qPCR
KM19	ATGTCATTGGCGATGAGACG	qPCR; 5′ *yycJ*; qPCR
KM20	GAATTAGGCGAGCGCTTAGG	qPCR; 5′ *spoVD*; qPCR
KM21	CTGTCCGAAAGCCGTAGTTG	qPCR; 3′ *spoVD*; qPCR
KM22	GCTCCGCATGGTCTATCGTG	qPCR; 5′ srfAA; qPCR
KM23	TTGCAAACGCTCCGCTTCTC	qPCR; 3′ srfAA; qPCR
KM4	CGGACTTGATGTTGAATCGTTTGGCGTTTCCC	5′ *yycJ*; PCR
KM5	GGAGAATACAAAGCGCCGACGACCGACATGG	5′ *srfAA*; PCR
KM76	AAAGGCTTTTGTAAATAAAG	In ICE*Bs1* near and amplifies towards the right end; qPCR
oJMA100	GGGTATACAATCATGGGTGATCGAG	in *yddN*, outside, but near and amplifies towards the right end of ICE*Bs1*; PCR [10], [11], [22]
oJMA102	TAATCTAAGCTTCACCTCCTCGTTAACTCAACTC	In ICE*Bs1* in *xis*, amplifies towards the left end of ICE*Bs1*; PCR [22]
oJMA141	CTTACTTTAGGTAAGTGGGCAGTTTGTGG	Overlaps the 3′ end of *int* in ICE*Bs1*; amplifies toward *attL* (into *trnS-leu2*); PCR [10]
oJMA207	GGATGAATTGTTTTAGTACCTAGATTTAGATGTC	In *kan*; near right end of ICE*Bs1* Δ(*rapI*-*phrI*)::*kan*; amplifies toward left end of ICE*Bs1*; inverse PCR, sequencing
oJMA227	ATATAAGCTTGCCTAGGCTCATTTTTATCATC	in ICE*Bs1*, upstream of ydcN; amplifies toward the left end of ICE*Bs1*; PCR
oJMA97	CTGTAAATTATGAATCTCAGATTGTTAATCCTGC	in ICE*Bs1* in *yddM*, amplifies toward right end of ICE*Bs1*; inverse PCR [10], [11], [22]

1 sequences are indicated 5′ to 3′.

2 the relevant location of each primer is indicated, along with how the primer was used. Primers to chromosomal regions are usually near the site of integration of ICE*Bs1*. The position, 5′ or 3′, in the indicated gene is relative to the direction of transcription of that gene, 5′ indicating extension in the same and 3′ indicating extension in the opposite direction as transcription. Left and right ends of ICE*Bs1* are as in Figure 1.

#### Backcross of ICE*Bs1* insertions

Seven of the 15 different insertions of ICE*Bs1* in secondary attachment sites were initially chosen for further study. These were first backcrossed into a strain cured of ICE*Bs1* (JMA222). Pxyl-*rapI* (*amyE*::{(Pxyl-*rap*) *spc*}) was introduced into these strains by transformation and selection for spectinomycin resistance using chromosomal DNA from strain MMB869. We verified that ICE*Bs1* was still at the original secondary attachment site using PCR with site-specific primers. The final strains from these crosses include: KM70 (*mmsA*::ICE*Bs1*), KM94 (*yvbT*::ICE*Bs1*), KM72 (*yrkM*::ICE*Bs1*), KM77 (*ykrP*::ICE*Bs1*), KM130 (*spoVD*::ICE*Bs1*), KM141 (*srfAA*::ICE*Bs1*), and KM132 (*yycJ*::ICE*Bs1*).

### Assays for excision and integration of ICE*Bs1*

#### Detecting excision from secondary insertions

Excision of ICE*Bs1* from a chromosomal attachment site creates an extrachromosomal ICE*Bs1* circle and an “empty” attachment site (also called “repaired chromosomal junction”). Each product was measured using specific primers for quantitative real time PCR (qPCR), using a LightCycler 480 Real-Time PCR system with Syber Green detection reagents (Roche), essentially as described [15]. Cells were grown in defined minimal medium with arabinose as carbon source. Products from excision were determined two hours after addition of xylose to induce expression of Pxyl-*rapI* to cause induction of ICE*Bs1* gene expression.

The amount of each empty attachment site was compared to a chromosomal reference gene, *cotF*, measured with primers CLO257-CLO258. The amount of empty attachment site from each of the secondary sites was normalized to strain JMA222, an ICE*Bs1*-cured strain that simulates 100% excision. Standard curves for qPCR with *cotF* and the repaired junction for each secondary insertion were generated using genomic DNA from JMA222. Primers (in parentheses) for empty secondary attachment sites were specific for: *yrkM* (CLO117-ABO17), *mmsA* (CLO109-ABO18), *yycJ* (KM18-KM19), *srfAA* (KM22-KM23), *spoVD* (KM20-KM21), *yvbT* (ABO14-ABO15), *ykrP* (KM154-KM16), and *attB* (CLO261-CLO262).

The amount of ICE*Bs1* circle that forms after excision from the chromosome was measured with primers AB019-CLO114. The amount of excised circle was compared to the chromosomal reference *cotF* (primers CLO257-CLO258), and normalized to the amount of excised circle from *attB* (strain CAL874). Standard curves for qPCR for *cotF* and the excised circle were generated using genomic DNA from RapI-induced CAL874. Primer sequences are presented in Table 3.

#### Detecting integration at *yrkM* in a pool of transconjugants

ICE*Bs1* was transferred from donor strain KM250 to recipient KM524 by conjugation, selecting for resistance to kanamycin and MLS antibiotics. Approximately 10^8^ transconjugants were collected from four separate conjugation experiments (done on filters placed on agar plates). Cells were washed off of all four filters with a total of 10 ml of minimal salts and aliquots of 0.2 ml were spread on selective plates to give ∼2×10^6^ transconjugants per plate. After overnight growth, plates (∼50) with the transconjugants were flooded with minimal salts, cells were scraped, collected, and transconjugants from all plates were pooled.

DNA was isolated from the pool of transconjugants and used as a template for qPCR with primers to detect the junction between *yrkM* and ICE*Bs1* (primers CLO116 and KM76). Values from this qPCR were compared to qPCR values for a reference gene (*cotF*). Values were normalized to a strain (KM72) that contains *yrkM*::ICE*Bs1* and represents 100% integration at *yrkM*. Values for *yrkM*::ICE*Bs1* in the pool of transconjugants were in the linear range of the qPCR and ≥3-fold above the background signal from the negative control (JMA222, which is cured of ICE*Bs1*). DNA used for standard curves was from strain KM72 (*yrkM*::ICE*Bs1*). The frequency of integration at *attB* was determined by qPCR with primers CLO273 and CLO264. Values were compared to *cotF* and normalized to a strain with ICE*Bs1* at *attB* (strain AG174 or CAL874). DNA used for standard curves was from AG174 or CAL874. The entire experiment was done twice with similar results.

#### Detecting integration at secondary sites after mating from a secondary site

Independent transconjugants, from donors with ICE*Bs1* at secondary attachment sites, were analyzed for the location of ICE*Bs1*. Sites analyzed and primers used included: *yrkM* (CLO116-CLO17 or oJMA141-CLO17); *mmsA* (CLO109-oJMA141); *yycJ* (CLO17-KM4); *srfAA* (oJMA141-KM5); and *attB* (CLO17-oJMA100). The presence of ICE*Bs1* in the transconjugants was verified using primers internal to ICE*Bs1* (oJMA102-oJMA22).

### Cell viability assays

Strains were grown in defined minimal medium with arabinose and expression of Pxyl-*rapI* was induced with 1% xylose at OD600 of 0.05. The number of colony forming units (CFU) was determined 3 hours after addition of xylose. For each strain, the number of CFU/ml 3 hrs after expression of Pxyl-*rapI* was compared to the number of CFU/ml without expression of Pxyl-*rapI*. All experiments were done at least twice.

### ß-galactosidase assays

Cells were grown and treated as described for viability assays. Samples were taken 3 hours after induction of Pxyl-*rapI*. All experiments were done at least twice. ß-galactosidase assays were done essentially as described [36], [37]. Specific activity is expressed as the (ΔA420 per min per ml of culture per OD600 unit)×1000.

### Modeling competition between cells with ICE*Bs1* in the primary attachment site versus cells with ICE*Bs1* in a secondary attachment site

We calculated the predicted population size P after G generations for cells in which ICE*Bs1* is integrated into a secondary attachment site, with an estimated fraction of dead cells, D. The estimate of dead cells is based on the fraction of cells in which ICE*Bs1* is excised during exponential growth, determined previously to be between 10^−5^ to 10^−4^. Population size P = P_0_•2^G^•(1−D)^G^, where P_0_ is the initial population size. The ratio R of the number of cells with ICE*Bs1* at *attB* to the number of cells with ICE*Bs1* in a secondary site is given by R = P_0_•2^G^ (for ICE*Bs1* in *attB* and assuming no killing upon induction)/P_0_•2^G^•(1−D)^G^ (for ICE*Bs1* in a secondary site). This equation reduces to R = 1/(1−D)^G^. This gives G = {log (1/R)}/log (1−D). For the number of cells with ICE*Bs1* in *attB* to be 10-fold greater than the number of cells with ICE*Bs1* in a secondary site (R = 10) and if the frequency of death if ∼10^−4^ for the secondary site insertions, then the number of generations to achieve R = 10 is: G = log(0.1)/log(0.9999) which is ∼23,000.

## References

[1] Wozniak RA, Waldor MK (2010) Integrative and conjugative elements: mosaic mobile genetic elements enabling dynamic lateral gene flow. Nat Rev Microbiol 8: 552–563. 20601965 10.1038/nrmicro2382

[2] Guglielmini J, Quintais L, Garcillan-Barcia MP, de la Cruz F, Rocha EP (2011) The repertoire of ICE in prokaryotes underscores the unity, diversity, and ubiquity of conjugation. PLoS Genet 7: e1002222. 21876676 10.1371/journal.pgen.1002222 PMC3158045

[3] Lee CA, Thomas J, Grossman AD (2012) The *Bacillus subtilis* conjugative transposon ICE*Bs1* mobilizes plasmids lacking dedicated mobilization functions. J Bacteriol 194: 3165–3172. 22505685 10.1128/JB.00301-12 PMC3370859

[4] Salyers AA, Shoemaker NB, Stevens AM, Li LY (1995) Conjugative transposons: an unusual and diverse set of integrated gene transfer elements. Microbiol Rev 59: 579–590. 8531886 10.1128/mr.59.4.579-590.1995 PMC239388

[5] Hochhut B, Waldor MK (1999) Site-specific integration of the conjugal *Vibrio cholerae* SXT element into *prfC* . Mol Microbiol 32: 99–110. 10216863 10.1046/j.1365-2958.1999.01330.x

[6] Roberts AP, Mullany P (2009) A modular master on the move: the Tn*916* family of mobile genetic elements. Trends Microbiol 17: 251–258. 19464182 10.1016/j.tim.2009.03.002

[7] Mullany P, Williams R, Langridge GC, Turner DJ, Whalan R, et al (2012) Behavior and target site selection of conjugative transposon Tn*916* in two different strains of toxigenic *Clostridium difficile* . Appl Environ Microbiol 78: 2147–2153. 22267673 10.1128/AEM.06193-11 PMC3302608

[8] Burrus V, Waldor MK (2004) Shaping bacterial genomes with integrative and conjugative elements. Res Microbiol 155: 376–386. 15207870 10.1016/j.resmic.2004.01.012

[9] Williams KP (2002) Integration sites for genetic elements in prokaryotic tRNA and tmRNA genes: sublocation preference of integrase subfamilies. Nucleic Acids Res 30: 866–875. 11842097 10.1093/nar/30.4.866 PMC100330

[10] Lee CA, Auchtung JM, Monson RE, Grossman AD (2007) Identification and characterization of *int* (integrase), *xis* (excisionase) and chromosomal attachment sites of the integrative and conjugative element ICE*Bs1* of *Bacillus subtilis* . Mol Microbiol 66: 1356–1369. 18005101 10.1111/j.1365-2958.2007.06000.x

[11] Auchtung JM, Lee CA, Monson RE, Lehman AP, Grossman AD (2005) Regulation of a *Bacillus subtilis* mobile genetic element by intercellular signaling and the global DNA damage response. Proc Natl Acad Sci U S A 102: 12554–12559. 16105942 10.1073/pnas.0505835102 PMC1194945

[12] Burrus V, Pavlovic G, Decaris B, Guedon G (2002) The ICE*St1* element of *Streptococcus thermophilus* belongs to a large family of integrative and conjugative elements that exchange modules and change their specificity of integration. Plasmid 48: 77–97. 12383726 10.1016/s0147-619x(02)00102-6

[13] Franke AE, Clewell DB (1981) Evidence for conjugal transfer of a *Streptococcus faecalis* transposon (Tn*916*) from a chromosomal site in the absence of plasmid DNA. Cold Spring Harb Symp Quant Biol 45 Pt 1: 77–80. 6271493 10.1101/sqb.1981.045.01.014

[14] Franke AE, Clewell DB (1981) Evidence for a chromosome-borne resistance transposon (Tn*916*) in *Streptococcus faecalis* that is capable of “conjugal” transfer in the absence of a conjugative plasmid. J Bacteriol 145: 494–502. 6257641 10.1128/jb.145.1.494-502.1981 PMC217299

[15] Lee CA, Babic A, Grossman AD (2010) Autonomous plasmid-like replication of a conjugative transposon. Mol Microbiol 75: 268–279. 19943900 10.1111/j.1365-2958.2009.06985.x PMC2905045

[16] Sitkiewicz I, Green NM, Guo N, Mereghetti L, Musser JM (2011) Lateral gene transfer of streptococcal ICE element RD2 (region of difference 2) encoding secreted proteins. BMC Microbiol 11: 65. 21457552 10.1186/1471-2180-11-65 PMC3083328

[17] Carraro N, Libante V, Morel C, Decaris B, Charron-Bourgoin F, et al (2011) Differential regulation of two closely related integrative and conjugative elements from *Streptococcus thermophilus* . BMC Microbiol 11: 238. 22024428 10.1186/1471-2180-11-238 PMC3234194

[18] Ramsay JP, Sullivan JT, Stuart GS, Lamont IL, Ronson CW (2006) Excision and transfer of the *Mesorhizobium loti* R7A symbiosis island requires an integrase IntS, a novel recombination directionality factor RdfS, and a putative relaxase RlxS. Mol Microbiol 62: 723–734. 17076666 10.1111/j.1365-2958.2006.05396.x

[19] Thomas J, Lee CA, Grossman AD (2013) A conserved helicase processivity factor is needed for conjugation and replication of an integrative and conjugative element. PLoS Genet 9: e103198. 10.1371/journal.pgen.1003198 PMC3542172 23326247

[20] Yoshida K, Yamaguchi M, Morinaga T, Kinehara M, Ikeuchi M, et al (2008) myo-Inositol catabolism in *Bacillus subtilis* . J Biol Chem 283: 10415–10424. 18310071 10.1074/jbc.M708043200

[21] Crooks GE, Hon G, Chandonia JM, Brenner SE (2004) WebLogo: a sequence logo generator. Genome Res 14: 1188–1190. 15173120 10.1101/gr.849004 PMC419797

[22] Auchtung JM, Lee CA, Garrison KL, Grossman AD (2007) Identification and characterization of the immunity repressor (ImmR) that controls the mobile genetic element ICE*Bs1* of *Bacillus subtilis* . Mol Microbiol 64: 1515–1528. 17511812 10.1111/j.1365-2958.2007.05748.x PMC3320793

[23] Lee CA, Grossman AD (2007) Identification of the origin of transfer (*oriT*) and DNA relaxase required for conjugation of the integrative and conjugative element ICE*Bs1* of *Bacillus subtilis* . J Bacteriol 189: 7254–7261. 17693500 10.1128/JB.00932-07 PMC2168444

[24] Berkmen MB, Lee CA, Loveday EK, Grossman AD (2010) Polar positioning of a conjugation protein from the integrative and conjugative element ICE*Bs1* of *Bacillus subtilis* . J Bacteriol 192: 38–45. 19734305 10.1128/JB.00860-09 PMC2798270

[25] Goranov AI, Katz L, Breier AM, Burge CB, Grossman AD (2005) A transcriptional response to replication status mediated by the conserved bacterial replication protein DnaA. Proc Natl Acad Sci U S A 102: 12932–12937. 16120674 10.1073/pnas.0506174102 PMC1200305

[26] Ireton K, Grossman AD (1994) A developmental checkpoint couples the initiation of sporulation to DNA replication in *Bacillus subtilis* . Embo J 13: 1566–1573. 8156995 10.1002/j.1460-2075.1994.tb06419.x PMC394986

[27] Cheo DL, Bayles KW, Yasbin RE (1991) Cloning and characterization of DNA damage-inducible promoter regions from *Bacillus subtilis* . J Bacteriol 173: 1696–1703. 1847907 10.1128/jb.173.5.1696-1703.1991 PMC207320

[28] Burrus V, Waldor MK (2003) Control of SXT integration and excision. J Bacteriol 185: 5045–5054. 12923077 10.1128/JB.185.17.5045-5054.2003 PMC181012

[29] Shimada K, Weisberg RA, Gottesman ME (1972) Prophage lambda at unusual chromosomal locations. I. Location of the secondary attachment sites and the properties of the lysogens. J Mol Biol 63: 483–503. 4552408 10.1016/0022-2836(72)90443-3

[30] Smits WK, Grossman AD (2010) The transcriptional regulator Rok binds A+T-rich DNA and is involved in repression of a mobile genetic element in *Bacillus subtilis* . PLoS Genet 6: e1001207. 21085634 10.1371/journal.pgen.1001207 PMC2978689

[31] Celli J, Trieu-Cuot P (1998) Circularization of Tn*916* is required for expression of the transposon-encoded transfer functions: characterization of long tetracycline-inducible transcripts reading through the attachment site. Mol Microbiol 28: 103–117. 9593300 10.1046/j.1365-2958.1998.00778.x

[32] Garrity DB, Zahler SA (1994) Mutations in the gene for a tRNA that functions as a regulator of a transcriptional attenuator in *Bacillus subtilis* . Genetics 137: 627–636. 8088508 10.1093/genetics/137.3.627 PMC1206022

[33] Ho SN, Hunt HD, Horton RM, Pullen JK, Pease LR (1989) Site-directed mutagenesis by overlap extension using the polymerase chain reaction. Gene 77: 51–59. 2744487 10.1016/0378-1119(89)90358-2

[34] Youngman P, Poth H, Green B, York K, Olmedo G, et al. (1989) Methods for Genetic Manipulation, Cloning, and Functional Analysis of Sporulation Genes in *Bacillus subtilis*. In: Smith I, Slepecky RA, Setlow P, editors. Regulation of Procaryotic Development. Washington, D.C.: ASM Press. pp. 65–87.

[35] Wach A (1996) PCR-synthesis of marker cassettes with long flanking homology regions for gene disruptions in *S. cerevisiae* . Yeast 12: 259–265. 8904338 10.1002/(SICI)1097-0061(19960315)12:3%3C259::AID-YEA901%3E3.0.CO;2-C

[36] Miller JH (1972) Experiments in Molecular Genetics. Cold Spring Harbor, N.Y.: Cold Spring Harbor Laboratory. xvi, 466 p.

[37] Jaacks KJ, Healy J, Losick R, Grossman AD (1989) Identification and characterization of genes controlled by the sporulation-regulatory gene *spo0H* in *Bacillus subtilis* . J Bacteriol 171: 4121–4129. 2502532 10.1128/jb.171.8.4121-4129.1989 PMC210181

[38] Glaser P, Frangeul L, Buchrieser C, Rusniok C, Amend A, et al (2001) Comparative genomics of *Listeria* species. Science 294: 849–852. 11679669 10.1126/science.1063447

[39] Perego M, Spiegelman GB, Hoch JA (1988) Structure of the gene for the transition state regulator, *abrB*: regulator synthesis is controlled by the *spo0A* sporulation gene in *Bacillus subtilis* . Mol Microbiol 2: 689–699. 3145384 10.1111/j.1365-2958.1988.tb00079.x

[40] Vandeyar MA, Zahler SA (1986) Chromosomal insertions of Tn*917* in *Bacillus subtilis* . J Bacteriol 167: 530–534. 3015878 10.1128/jb.167.2.530-534.1986 PMC212921

[41] Smith BT, Grossman AD, Walker GC (2001) Visualization of mismatch repair in bacterial cells. Mol Cell 8: 1197–1206. 11779496 10.1016/s1097-2765(01)00402-6

